# A Polysaccharide From *Eupolyphaga sinensis* Walker With Anti-HBV Activities *In Vitro* and *In Vivo*

**DOI:** 10.3389/fphar.2022.827128

**Published:** 2022-03-03

**Authors:** Xue Zhang, Huiling Su, Haifei Yu, Jialu Ding, Wanyu Deng, Bo Qin, Changlin Zhou, Jie Dou, Min Guo

**Affiliations:** ^1^ State Key Laboratory of Natural Medicines, School of Life Science and Technology, China Pharmaceutical University, Nanjing, China; ^2^ College of Life Science, Shangrao Normal University, Shangrao, China; ^3^ Department of Biliary Pancreatic Surgery, Sun Yat-sen Memorial Hospital, Sun Yat-sen University, Guangzhou, China; ^4^ Shaoxing Women and Children’s Hospital, Shaoxing, China

**Keywords:** ESPS, anti-HBV activity, Toll-like receptor, IFN signaling system, pro-inflammatory cytokine, HNF4α

## Abstract

Hepatitis B virus (HBV) infection remains a major global threat to human health worldwide. Recently, the Chinese medicines with antiviral properties and low toxicity have been a concern. In our previous study, *Eupolyphaga sinensis* Walker polysaccharide (ESPS) has been isolated and characterized, while its antiviral effect on HBV remained unclear. The anti-HBV activity of ESPS and its regulatory pathway were investigated *in vitro* and *in vivo*. The results showed that ESPS significantly inhibited the production of HBsAg, HBeAg, and HBV DNA in the supernatants of HepG2.2.15 in a dose-dependent manner; HBV RNA and core protein expression were also decreased by ESPS. The *in vivo* studies using HBV transgenic mice further revealed that ESPS (20 and 40 mg/kg/2 days) significantly reduced the levels HBsAg, HBeAg, and HBV DNA in the serum, as well as HBV DNA and HBV RNA in mice liver. In addition, ESPS activated the Toll-like receptor 4 (TLR4) pathway; elevated levels of IFN-β, TNF-α, and IL-6 in the serum were observed, indicating that the anti-HBV effect of ESPS was achieved by potentiating innate immunity function. In conclusion, our study shows that ESPS is a potential anti-HBV ingredient and is of great value in the development of new anti-HBV drugs.

## Introduction

Hepatitis B virus (HBV) infection remains a major and serious global health problem, and with 257 million chronically infected people worldwide, HBV infection often results in acute or chronic infection and contributes to a high risk of severe liver disease including liver failure, cirrhosis, hepatocellular carcinoma (HCC), or death (Yuen et al., 2018; Likhitsup et al., 2019; Carey et al., 2020). Due to the lack of drugs with sustained virological response, the number of hepatitis B patients is increasing every year (Vittal and Ghany, 2019; Higashi-Kuwata et al., 2021). In recent decades, more and more evidence show that HBV causes different degrees of virus replication and liver inflammation under the dual effect of host–virus, leading to congenital immune dysfunction (Luangsay et al., 2015). Therefore, virological treatment is the basis of chronic hepatitis B and other related severe liver diseases (Revill et al., 2016). However, the effect of antiviral therapy is limited. Nucleoside analogues (NAS) need to be taken for a long time; interferon-α (IFN) has inevitable side effects (Zoulim et al., 2016; Lopatin, 2019). Therefore, the development of alternative strategies for HBV is imminent. At present, more and more attention has been paid to the investigation of polysaccharides, and they have been reported to have excellent anti-HBV activity (Lin et al., 2016; Yang et al., 2018).

Usually, natural products have novel structure and many kinds of pharmacological activities, which is a huge reservoir for the development of new anti-HBV drugs (Chen and Huang, 2018). *Eupolyphaga sinensis* Walker (ESW) belongs to the family Corydiidae (Blattodea), which is a kind of important insect and widely distributed in China (Zhan et al., 2016). In traditional medicine, ESW enhanced immune response and promoted blood circulation by removing blood stasis, so it was widely used in clinical natural healthcare products (Tang et al., 2010). In addition, ESW also treated a variety of diseases, including bone injury, cancer, and immune-related diseases (Wu et al., 2013). However, the antiviral effect was still unclear.

Recently, a new water-soluble polysaccharide, named ESPS, was separated from ESW in our laboratory. The primary chemical structure was studied with an average molecular weight of 21.4 kDa, and containing rhamnose (7.4%), fucose (3.1%), arabinose (13.9%), xylose (9.3%), glucose (39.7%), and galactose (26.5%) (Xie et al., 2020). We applied for a patent on its structure and use; the patent publication number is CN110894244A. Our previous studies showed that ESPS exhibited notable effects on immunity, such as restoring and improving the phagocytic ability of mouse macrophages, and inhibiting tumor growth (Xie et al., 2020). In this study, the hepatoma cell line HepG2.2.15 and C57BL/6J HBV-transgene mice were used as experimental models *in vitro* and *in vivo*. The results displayed that ESPS significantly inhibited HBV antigen, HBV DNA, and HBV core protein formation through TLR4 pathway. All data showed that ESPS has therapeutic potential for HBV.

## Materials and Methods

### Chemicals and Reagents

ESPS was prepared in our laboratory (Xie et al., 2020). LPS and lamivudine were purchased from Sigma (St. Louis, MO, USA). Cell counting kit-8 (CCK8), Trizol agent, and dual luciferase reporter gene kit were purchased from Nanjing Novozan Biotechnology Co., Ltd. (Jiangsu, China). Interferon-β (IFN-β) ELISA kit was purchased from Elabscience (Wuhan, China). Dulbecco modified Eagle’s medium (DMEM) and fetal bovine serum (FBS) were obtained from Gibco (Grand Island, New York, USA). Penicillin and streptomycin were purchased from Beyotime Biotechnology (Shanghai, China). PrimeScript RT reagent kit and TB premix ex Taq were purchased from Takara (Liaoning, China). Tris-saturated phenol was purchased from Solebo Technology Co., Ltd. (Beijing, China). The ELISA kits for HBsAg and HBeAg were purchased from Kehua Bioengineering Co., Ltd. (Shanghai, China). Qiaamp DNA blood mini kit was purchased from Qiagen. TAK242 was purchased from MCE (New Jersey, USA). All other reagents used were analytically pure.

### Cells and Animals

The hepatoma cell line HepG2.2.15 was purchased from ATCC (USA), cultured in DMEM medium containing 10% FBS, 100 U/ml penicillin, and 100 μg/ml streptomycin and incubated in an atmosphere at 37°C and 5% carbon dioxide.

Male C57BL/6J mice, weighing 21 ± 2 g, were purchased from Weitong Lihua Laboratory Animal Technology Co., Ltd. (Zhejiang, China), which can freely access to food and water under the conditions of temperature (22–25°C), humidity (55–70%), and 12-h light/dark cycle according to the China Pharmaceutical University Animal Care and Use Guidelines (Nanjing, China).

After 1-week adaptation, 8 μg pAAV-1.2HBV plasmid was injected into the tail vein of C57BL/6J mice by high-pressure hydrodynamic method within 5–8 s (Wu et al., 2014). Twenty-four hours later, blood was taken from the eye frame and the serum levels of HBsAg, HBeAg, and HBV DNA were detected. Then the mice were randomly divided into five groups (N, control group; M, model group; LP, 20 mg/kg/2 days ESPS treatment group; HP, 40 mg/kg/2 days ESPS treatment group; AP, 30 mg/kg/2 days 3TC treatment group). Animals got PBS and ESPS by intravenous injection, and 3TC by gavage. The administration volume was 10 ml/kg, and the administration was continuous for 20 days. Serum was collected at indicated times. At the end of the experiment, the mice were sacrificed by neck dissection, the abdominal cavities were dissected, and the fresh liver were taken out and stored. During the administration, the hair, diet, and mental and activity status of mice were observed, and the weight of mice was measured.

### Cell Cytotoxicity and Viability Assays

HepG2.2.15 or Huh7 cells were seeded in a 96-well plate with 1 × 10^4^ cells per well, cultured at 37°C with 5% CO_2_. The cytotoxicity of ESPS on HepG2.2.15 and Huh7 cells was evaluated by CCK8 method (Long et al., 2015). In short, ESPS were diluted in the medium (DMEM with 10% FBS) to the concentrations of 50, 100, 200, 400, and 800 μg/ml, and treated HepG2.2.15 and Huh7 cells at indicated concentrations for 48 h. After 48 h of ESPS treatment, 100 μl DMEM containing 10 μl CCK8 was replaced to each well and incubated for another 1 h at 37°C. Then, the absorbance at 450 nm was measured by using a microplate reader (Bio-Rad, USA). Cell viability was expressed as a percentage of untreated control group.

### q-PCR

Total RNA was extracted from mouse liver homogenate or human hepatoma cells by Trizol lysis method, and used for synthesis of cDNA with PrimeScript RT reagent Kit according to instruction. Total DNA was extracted from mouse liver homogenate or human hepatoma cells with Tris-saturated phenol. Total DNA was extracted from mouse serum or culture supernatant of human hepatoma cells with Qiaamp DNA blood kit.

Then expression levels of the following genes were analyzed with quantitative real-time PCR (qPCR) assays using TB Premix Ex Taq in the CFX96 Real-time fluorescence PCR system (Bio-Rad, USA). The primers of forward and reverse sequences from 5′ to 3′ were respectively shown in Table 1. The mRNA data were calibrated to GAPDH; all mRNA and DNA data were calculated via the method of ΔΔCt, which could figure out the expression level of mRNA and DNA equal to 2^−ΔΔCt^.

**TABLE1:** Gene sequence

Gene	Sequence
Human GAPDH-F	5′-AAA TCA AGT GGG GCG ATG CTG-3′
Human GAPDH-R	5′-GCA GAG ATG ATG ACC CTT TTG-3′
HBV core-associated DNA-F	5′-ACC AAT CGC CAG TCA GGA AG-3′
HBV core-associated DNA-R	5′-ACC AGC AGG GAA ATA CAG GC-3′
pg RNA-F	5′-CTG GGT GGG TGT TAA TTT GG-3′
pg RNA-R	5′-TAA GCT GGA GGA GTG CGA AT-3′
Total RNA-F	5′-TCA CCA GCA CCA TGC AAC-3′
Total RNA-R	5′-AAG CCA CCC AAG GCA CAG-3′
Human TLR4-F	5′-TCT TGG TGA AGT TGA ACG G-3
Human TLR4-R	5′-GCC ACA CCG GGA ATA A-3′
Human TLR2-F	5′-CTG TGC TCT GTT CCT GCT GA-3′
Human TLR2-R	5′-GAT GTT CCT GCT GGG AGC TT-3′
Human IFN-α-F	5′-CCA GTT CCA GAA GGC TCC AG-3′
Human IFN-α-R	5′-CTG CTC TGA CAA CCT CCC AG-3′
Human IFN-β-F	5′-GTG AGG AAA TAC TTC CAA AGA ATC AC-3′
Human IFN-β-R	5′-GTG AGG AAA TAC TTC CAA AGA ATC AC-3′
Human MxA-F	5′-CTC CGA CAC GAG TTC CAC AA-3′
Human MxA-R	5′-GGC TCT TCC AGT GCC TTG AT-3′
Human OAS-1-F	5′-GAA GGC AGC TCA CGA AAC-3′
Human OAS-1-R	5′-TTC TTA AAG CAT GGG TAA TTC-3′
Human TNF-α-F	5′-ATC TTC TCG AAC CCC GAG TGA-3′
Human TNF-α-R	5′-CGG TTC AGC CAC TGG AGC T-3′
Human IL-6-F	5′-TTC GGT CCA GTT GCC TTC TC-3′
Human IL-6-R	5′-CAG CTC TGG CTT GTT CCT CA-3′
Human HNF4α-F	5′-CGA AGG TCA AGC TAT GAG GAC A-3′
Human HNF4α-R	5′-ATC TGC GAT GCT GGC AAT CT-3′
Mouse TLR4-F	5′-ATG GCA TGG CTT ACA CCA CC-3′
Mouse TLR4-R	5′-GAG GCC AAT TTT GTC TCC ACA-3′
Mouse TLR2-F	5′-CTC TTC AGC AAA CGC TGT TCT-3′
Mouse TLR2-R	5′-GGC GTC TCC CTC TAT TGT ATT G-3′
Mouse MxA-F	5′-GAC CAT AGG GGT CTT GAC CAA-3′
Mouse MxA-R	5′-AGA CTT GCT CTT TCT GAA AAG CC-3′
Mouse OAS-1-F	5′-GGC CTC TAA GGG GGT CAA G-3′
Mouse OAS-1-R	5′-CTG GCA GCA CGT CAA ACT TC-3′
Mouse TNF-α-F	5′-CCC TCA CAC TCA GAT CAT CTT CT-3′
Mouse TNF-α-R	5′-GCT ACG ACG TGG GCT ACA G-3′
Mouse IL-6-F	5′-ACT TCC ATC CAG TTG CCT TCT TGG-3′
Mouse IL-6-R	5′-TTA AGC CTC CGA CTT GTG AAG TGG-3′

### Western Blot

HepG2.2.15 cells were seeded in a 6-well plate with 2 × 10^5^ cells per well and cultured at 37°C with 5% CO_2_, treated with ESPS (50, 100, 200, and 400 μg/ml) or 3TC (2.45 μg/ml) or LPS (1 μg/ml)for 6 days or 16 h. And then, HepG2.2.15 cells were lysed in Ripa (Bestbio, Shanghai, China) containing 1% PMSF (Sigma, USA) and 0.01% phosphatase inhibitor (TIANGEN, Beijing, China). The loading buffer was added into the homogenate, accounting for 20%. After boiling at 100°C for 10 min, the protein extracts were electrophoretically separated on 10% SDS-PAGE gel and then transferred to a polyvinylidene fluoride (PVDF) membrane at 340 mA for 90 min. Then, the membranes were blocked with 5% BSA (Action-award, Guangzhou, China) for 2 h, incubated with anti-TLR4 (Catalog No. GTX75742; GeneTex), anti-TLR2 (Catalog No. GTX00996; GeneTex), anti-p-P65 (Catalog No. GTX133899; GeneTex), anti-p65 (Catalog No. GTX102090; GeneTex), anti-IRF3 (Catalog No. GTX84287; GeneTex), anti-p-IRF3 (Catalog No. GTX130422; GeneTex), anti-HBc (Catalog No. SC-23945; Santa Cruz), anti-HBs (Catalog No. SC-53299; Santa Cruz), anti-HB pol (Catalog No. SC-81590; Santa Cruz), anti-HNF4α (Catalog No. GTX01036; GeneTex), and anti-β-actin (Catalog No. SC-47778, Santa Cruz) at 4°C overnight and probed with secondary antibody (ABclonal, Hubei, China; Rayantibody, Beijing, China) conjugated with horseradish peroxidase at 37°C for 2 h. Finally, the bands of protein on the membrane were visualized by 30% acrylamide and DAB Immunohistochemistry Color Development Kit (Vazyme, Nanjing, China) on a Bio-Imaging system, and the band intensities of proteins were quantified using ImageJ and normalized to β-actin levels.

### Detection of HBsAg, HBeAg and IFN-β in HepG2.2.15 Cells and Mice Serum

The levels of HBsAg, HBeAg, and IFN-β in HepG2.2.15 cells and mice serum were determined using ELISA kits according to the manufacturer’s instructions.

### Immunohistochemical

For immunohistochemical investigation (Fang et al., 2019), a portion of the right lobe of liver was removed, fixed in 4% paraformaldehyde, and embedded in paraffin. After pathological sectioning, liver histopathologic changes were examined by light microscopy. A total of 15 tissue sections were analyzed.

### Statistical Analysis

All of the experimental data were presented as the mean ± SD. Also, statistical analysis was calculated using GraphPad Prism 8.0. Comparison between groups was performed using one-way ANOVA, while considering a *p*-value <0.05 as statistically significant.

## Results

### Effect of ESPS on HBV Activity *in vitro*

**FIGURE 1:**
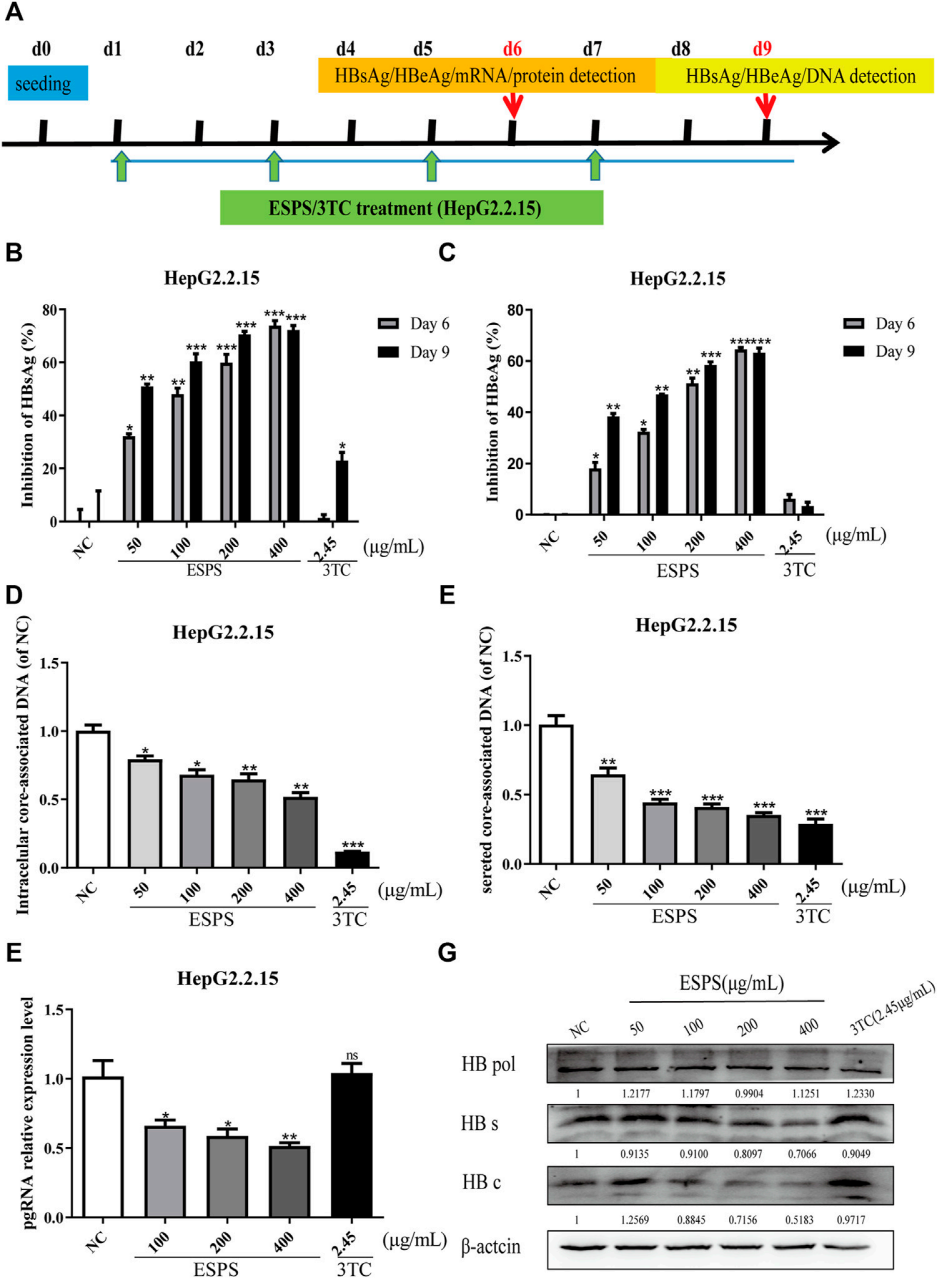
Inhibitory effects of ESPS on HBV *in vitro*. **(A)** Experimental process of inhibiting HBV by ESPS *in vitro*. HepG2.2.15 cells were seeded at day 0, after treatment with ESPS at indicated concentrations, HBsAg **(B)**, HBeAg **(C)** in the supernatant of HepG2.2.15 cells were measured by ELISA on the sixth and ninth days. qPCR was used to detect the levels of intracellular core-associated DNA **(D)**, secreted core-associated DNA **(E)** on the ninth day, and HBV mRNA **(F)** on the sixth day. Western blotting analysis was used to detect the levels of HBV protein **(G)** on the sixth day. 3TC was used as positive control. Values are means ± SD (*n* = 3). *p < 0.05, **p < 0.01, ***p < 0.001 *vs*. the normal control group.

HepG2.2.15 is recognized as a cell model for screening and evaluating anti-HBV drugs *in vitro* (Zhang et al., 2016). We wondered whether the ESPS had an anti-HBV effect. The maximum non-toxic concentration of ESPS was about 400 μg/ml; the dose of ESPS for subsequent experiment was under 400 μg/ml (Supplementary Figures S1A,B). As shown in Figure 1A, to determine the inhibitory effect of ESPS on viral replication, cells were incubated with ESPS or 3TC for 6/9 days. Then, samples were collected at indicated time points. Compared with normal control group, HBsAg, HBeAg, and extracellular HBV DNA were significantly inhibited by ESPS (*p* < 0.001) (Figures 1B–E), while after treatment with ESPS (400 μg/ml), intracellular HBV DNA, HBV pgRNA, and HBV total RNA decreased by 48.18, 48.51, and 47.91%, respectively (*p* < 0.01) (Figure 1F). The intracellular core protein and the HBsAg expression decreased by 48.17 and 29.34% after treatment with ESPS (400 μg/ml), respectively. However, the level of polymerase protein showed no significant difference (Figure 1G). Therefore, ESPS had anti-HBV activity *in vitro*, but the anti-HBV mechanism was different from that of nucleoside analogues.

### ESPS Inhibit HBV Replication Through TRL4

**FIGURE 2:**
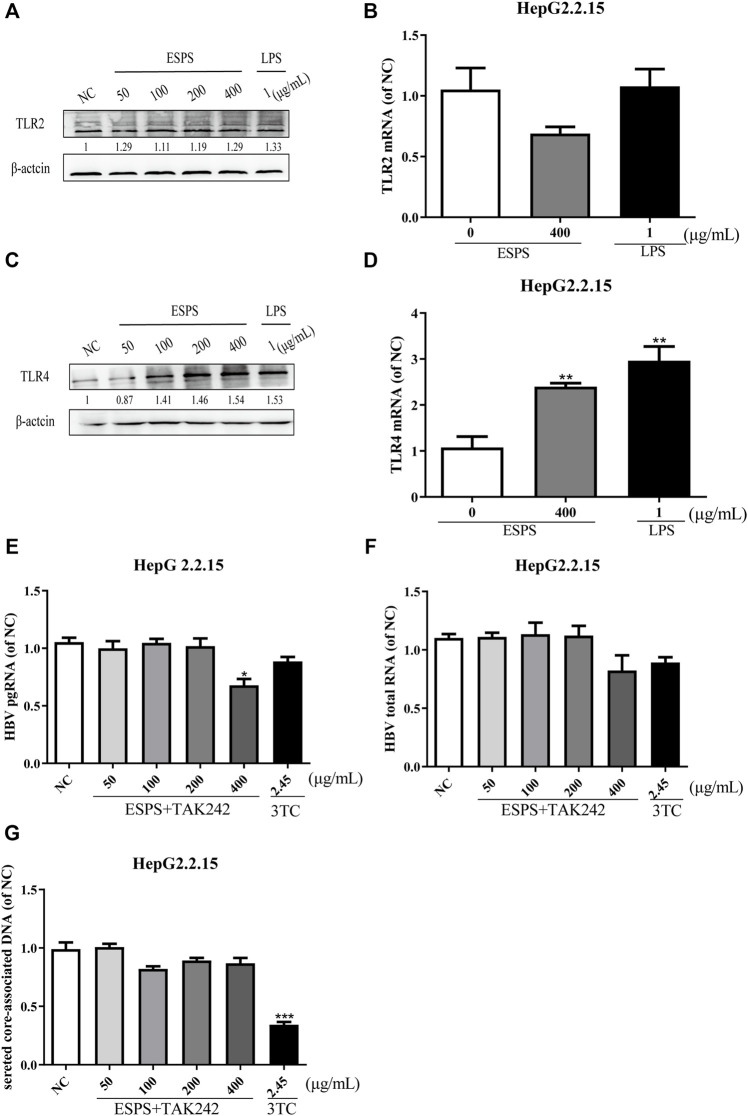
ESPS inhibit HBV replication through TRL4 pathway. HepG2.2.15 cells were treated with indicated compounds for 16 h; the protein levels of TLR2 and TLR4 were evaluated by western blotting analysis **(A,C)**. Then the mRNA level of TLR2 and TLR4 was evaluated by Q-PCR **(B,D)**. HepG2.2.15 cells were treated with the inhibitor of TLR4 (TAK242) and ESPS, qPCR was used to evaluate the HBV pgRNA **(E)** and total mRNA **(F)** after 6 days, and HBV core-associated DNA **(G)** was evaluated after 9 days. Values are means ± SD (*n* = 3). *p < 0.05, **p < 0.01, ***p < 0.001 *vs*. control group.

To elucidate the molecular mechanisms of ESPS on anti-HBV action, we first determined the classic polysaccharide receptor, Toll-like receptor 2 (TLR2) and 4 (TLR4) expression in HepG2.2.15 cells. The mRNA and protein levels of TLR2 were not changed (*p* > 0.05) (Figures 2A,B), while the expression of TLR4 were improved in a dose-dependent manner (*p* < 0.05) (Figures 2C,D) after treatment with various concentrations of ESPS (50, 100, 200, 400 μg/ml). To further explore whether TLR4 is a potential target of ESPS, the inhibitor of TLR4 (TAK242) has been used to block the activation of TLR4. As shown in Figures 2E–G, the inhibitory effects of ESPS (50, 100, 200, 400 μg/ml) on HBV pgRNA, HBV total mRNA, and secreted core associated DNA were blocked by TAK242. Thus, TLR4 may be the potential target of ESPS on HepG2.2.15 cells.

### Activation of Interferon Signaling Pathway by ESPS in HepG2.2.15 Cells

**FIGURE 3:**
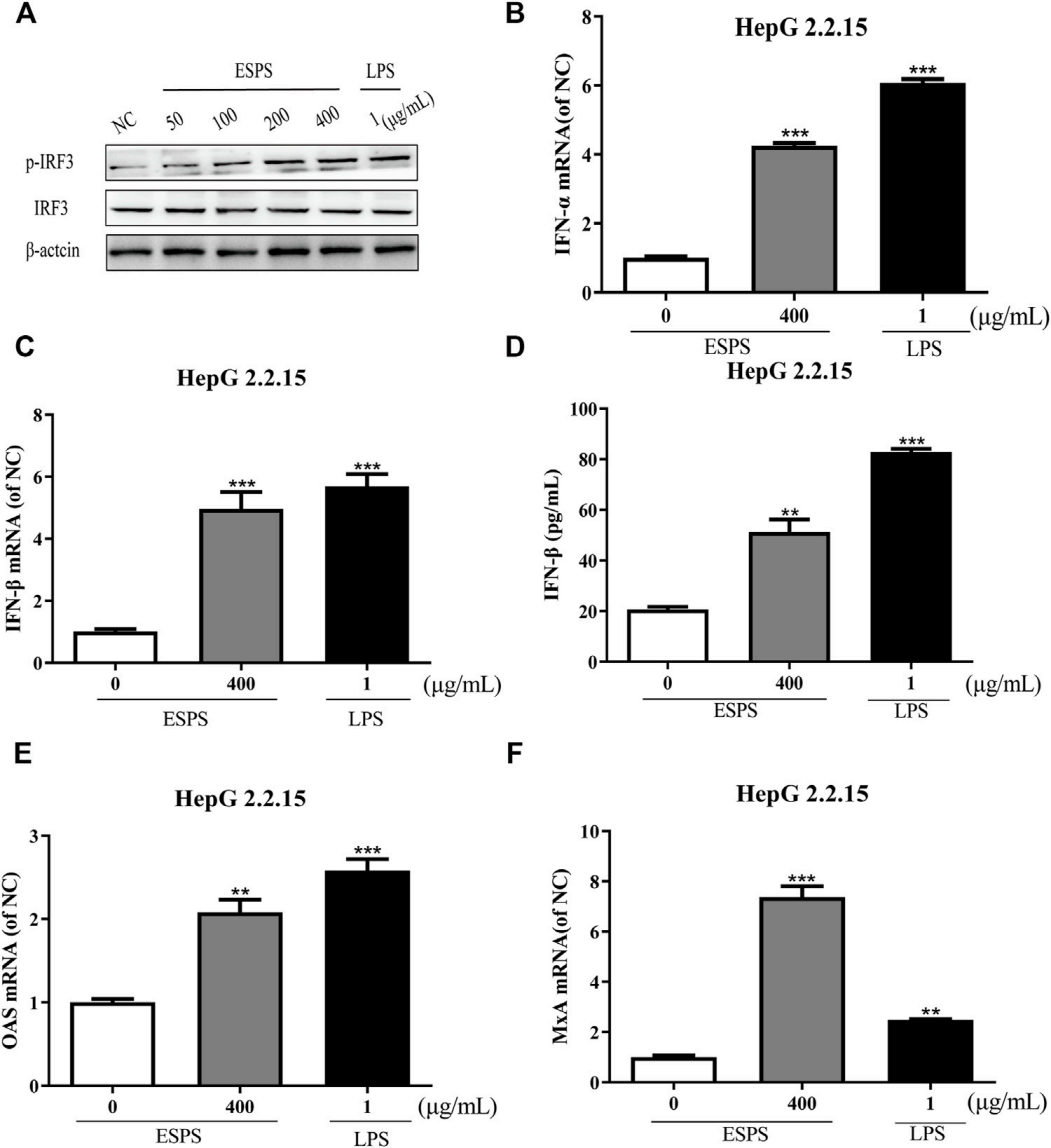
Activation of interferon signaling pathway by ESPS in HepG2.2.15 cells. After treatment with ESPS at indicated concentrations, Western blotting analysis was used to detect the levels of IRF3 or p-IRF3 protein; β-actin protein was used as loading control **(A)**. qPCR was used to detect the levels of intracellular IFN-α **(B)**, IFN-β **(C)**, OAS **(E)**, and MxA **(F)**. Secreted IFN-β **(D)** was detected by ELISA. LPS was used as positive control. Values are means ±SD (*n* = 3). **p < 0.01, ***p < 0.001 *vs*. the normal control group.

The contribution of host innate immunity on clearance of HBV has been explored, and antiviral mediators such as interferon (IFN) and other immunoglobulin factors are considered to be the first line of antiviral immunity (Wu et al., 2009; Belloni et al., 2012; Niu et al., 2018; Wu et al., 2018). We wonder whether ESPS inhibits HBV replication through TLR-4/IRF3/INF pathway; lipopolysaccharide (LPS) as an agonist for TLR4 was used as positive control. First, we tested whether ESPS affected the protein expression of IRF3 in HepG2.2.15 cells. Western blotting analysis showed that the level of total IRF3 protein remained unchanged, while p-IRF3 was promoted in a dose-dependent manner (Figure 3A). Compared with the control group, the transcription levels of IFN-α and IFN-β were increased by 4.25-fold and 4.96-fold, respectively (Figures 3B,C). In addition, ELISA showed that the level of IFN-β in supernatant was increased by 1.48-fold after ESPS (400 μg/ml) treatment (Figure 3D). The transcriptional level of interferon-stimulated genes (ISGs), such as OAS and MxA, was increased by 2.08-fold and 7.37-fold, respectively (Figures 3E,F). Therefore, ESPS may inhibit HBV replication *via* enhancement of the interferon system *in vitro*.

### NF-κB and ERK Signaling Pathways were Involved in the Anti-HBV Actions of ESPS *in vitro*

NF-κB is an important regulator of inflammation, immunity, or apoptosis and other pathological processes (Zeisel et al., 2015; Li et al., 2019; Namineni et al., 2020). Activation of NF-κB has been reported to increase the production of pro-inflammatory cytokines. Then pro-inflammatory cytokines can activate ERK to play a direct antiviral function (Takeuchi and Akira, 2010). Thus, the effect of ESPS on NF-κB signaling pathway was explored.

**FIGURE 4:**
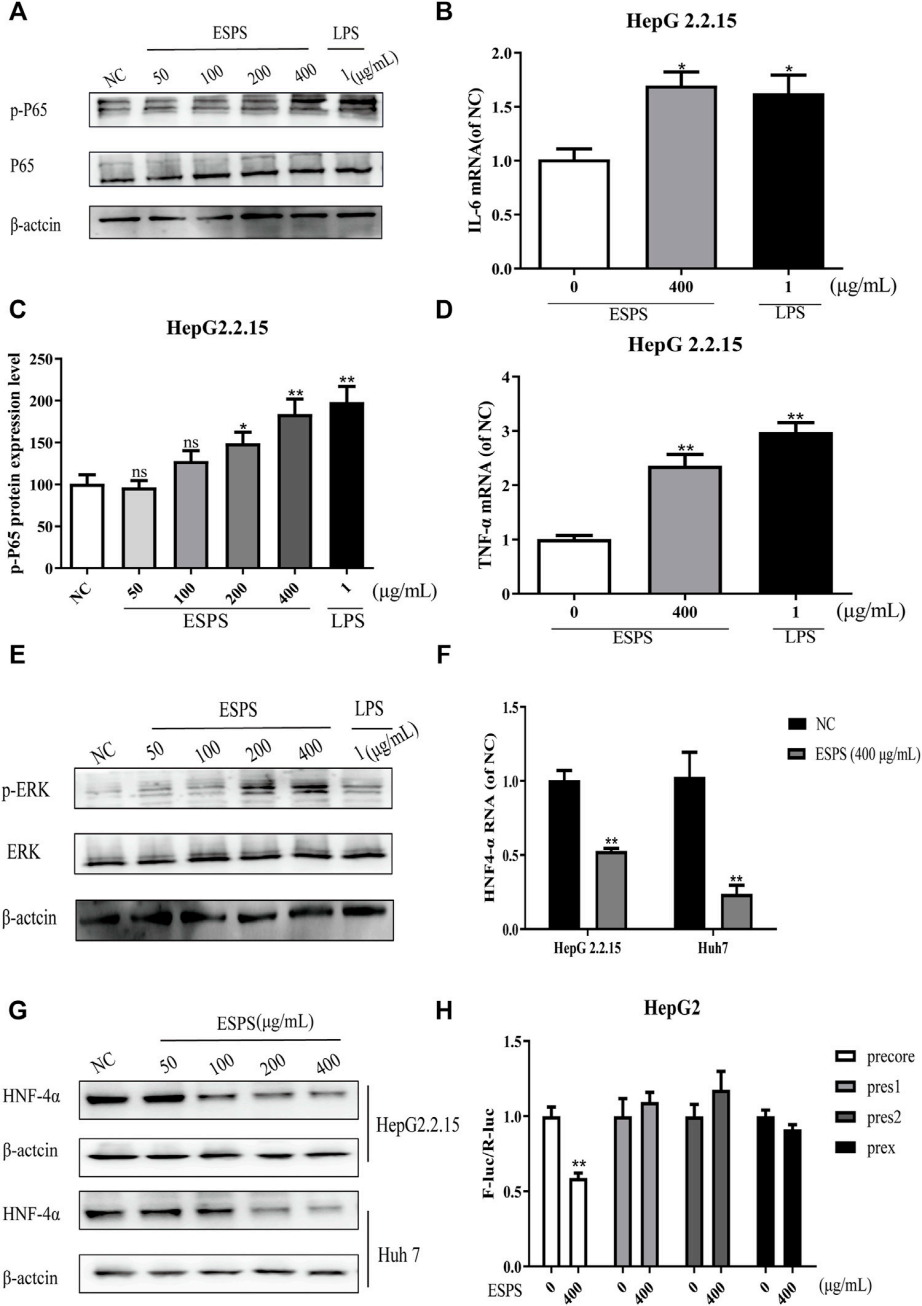
Effects of ESPS on NF-κB and ERK signaling pathways *in vitro*. HepG2.2.15 cells were treated with indicated compounds for 16 h; the phosphorylation and total expression of NF-κB proteins were evaluated by Western blotting analysis **(A)**. Quantification of immunoblot for the ratio of p-NF-κB or total NF-κB protein to β-actin protein, respectively. The ratio for non–drug-treated control cells was assigned values of 1.0 **(C)**. The mRNA levels of IL-6 **(B)** and TNF-α **(D)** were evaluated by qPCR. The phosphorylation and total expression of ERK1/2 proteins were evaluated by Western blotting analysis **(E)**. The protein level **(F)** and mRNA level **(G)** of HNF4α were detected by Western blotting and qPCR analysis, respectively. After HBV promoter was transfected, HepG2 cells were treated with ESPS. Then the promoter activity was analyzed by double luciferase **(H)**. Values are means ± SD (*n* = 3). *p < 0.05, **p < 0.01 *vs*. control group.

The results showed that the level of phosphorylated NF-κB was significantly increased after 16-h treatment of ESPS (50, 100, 200, 400 μg/ml), compared with negative control group, while the total level of NF-κB had no significant change. The positive control LPS also increased the phosphorylated level of NF-κB (Figures 4A,C). As shown in Figures 4B,D, the expression levels of IL-6 and TNF-α in HepG2.2.15 cells were significantly elevated by ESPS (400 μg/ml), and they were 0.70-fold and 1.36-fold higher than those of non-treated cells (*p* < 0.05; *p* < 0.01), respectively.

ERK signaling pathway was reported to be able to downregulate the expression of hepatocyte nuclear factor 4α (HNF4α), which is a very important factor for HBV transcription (Kawai and Akira, 2011). Thus, we evaluated the effects of ESPS on ERK pathway. The western blotting analysis showed that the levels of phosphorylated ERK1/2 in HepG2.2.15 cells were significantly upregulated after 16-h treatment of ESPS (50, 100, 200, 400 μg/ml) compared with the negative control group (Figure 4E). Then, we further evaluated the effects of ESPS on HNF4α. The western blotting and qPCR analysis showed that the levels of HNF4α in HepG2.2.15 or HepG2cells were significantly downregulated after 16-h treatment of ESPS, compared with the non–drug-treated control group (*p* < 0.01) (Figures 4F,G). As expected, HBV core promoter activity was downregulated by ESPS in HepG2.2.15 cells (*p* < 0.01) (Figure 4H). Therefore, ESPS may inhibit HBV replication *via* NF-κB/ERK/HNF4α axis.

### Effect of ESPS on HBV Activity *in vivo*

**FIGURE 5:**
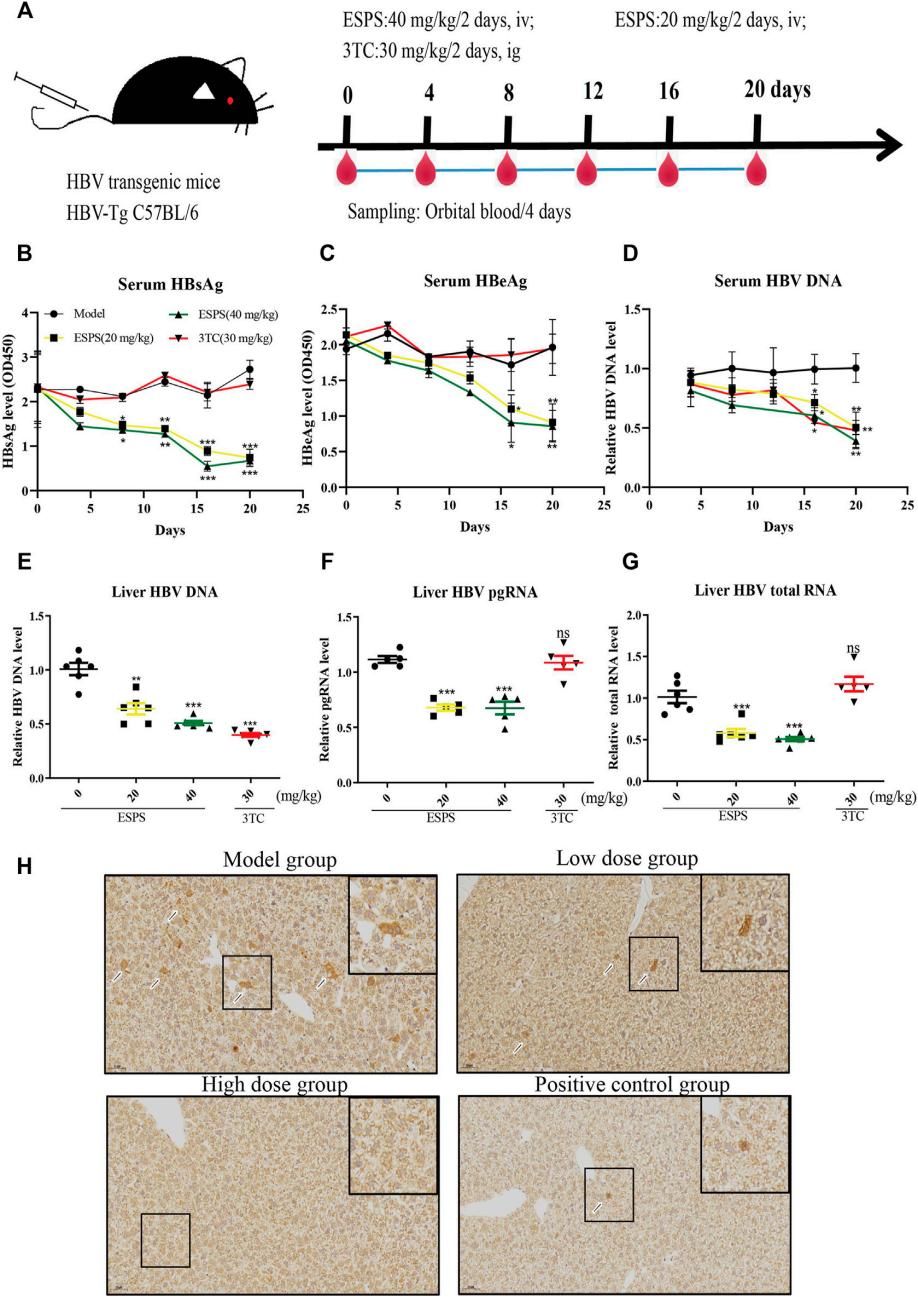
Inhibitory effects of ESPS on HBV *in vivo*. **(A)** Experimental process for inhibiting of HBV by ESPS *in vivo*. C57BL/6J mice were injected with plasmid pAAV-HBV1.2 *via* HI. On day D-0, the mice were divided into four groups according to the results of HBsAg detection. The blood was taken every 4 days and weighed, and the liver tissue was taken out on day 20. After treatment with ESPS at indicated times, ELISA was used to detect the levels of serum HBsAg **(B)** and HBeAg **(C)**. The levels of core-associated DNA in the serum **(D)** and in the liver tissue **(E)**, HBV pgRNA **(F)**, and HBV total mRNA **(G)** in the liver issue of mice were detected by qPCR; HBcAg in the right lobe of model group (PBS treatment group), low-dose group (20 mg/kg/2 days ESPS treatment group), high-dose group (40 mg/kg/2 days ESPS treatment group), and positive control group (30 mg/kg/2 days 3TC treatment group) **(H)** were detected by immunohistochemistry on day 20. 3TC was used as positive control. Values are means ± SD (*n* = 6). *p < 0.05, **p < 0.01, ***p < 0.001 *vs*. the model control group.

We further investigated the influence of ESPS on HBV replication in a hydrodynamic injection (HI) mouse model. After injection for 24 h, HBsAg-positive mice were randomly divided into four groups, including PBS (model group), low-dose group (20 mg/kg/2 days ESPS treatment group), high-dose group (40 mg/kg/2 days ESPS treatment group), and positive control group (30 mg/kg/2 days 3TC treatment group) (Figure 5A). Consistent with the results *in vitro*, compared with the PBS group, the level of serum HBsAg decreased by about 35% at day 8 in the low-dose and high-dose group; the level of serum HBeAg decreased by about 30% at day 12 in the low-dose and high-dose groups (Figures 5B,C). In the high-dose group, the level of serum HBV DNA decreased to 40% at day 16, which was equivalent to that of the positive control group (Figure 5D). Similarly, ESPS treatment reduced the level of HBV DNA, HBV pgRNA, and HBV total mRNA in liver at day 20 (Figures 5E,F). In addition, immunohistochemistry results showed that HBcAg in the right lobe of liver was significantly decreased after ESPS treatment (Figure 5H). Moreover, no significant difference in body weight was observed in all mice (data not shown). The hair, diet, and mental and activity status of mice were all normal in the healthy controls.

### Influence on the Innate Immunomodulatory Activity by ESPS *in vivo*

**FIGURE 6:**
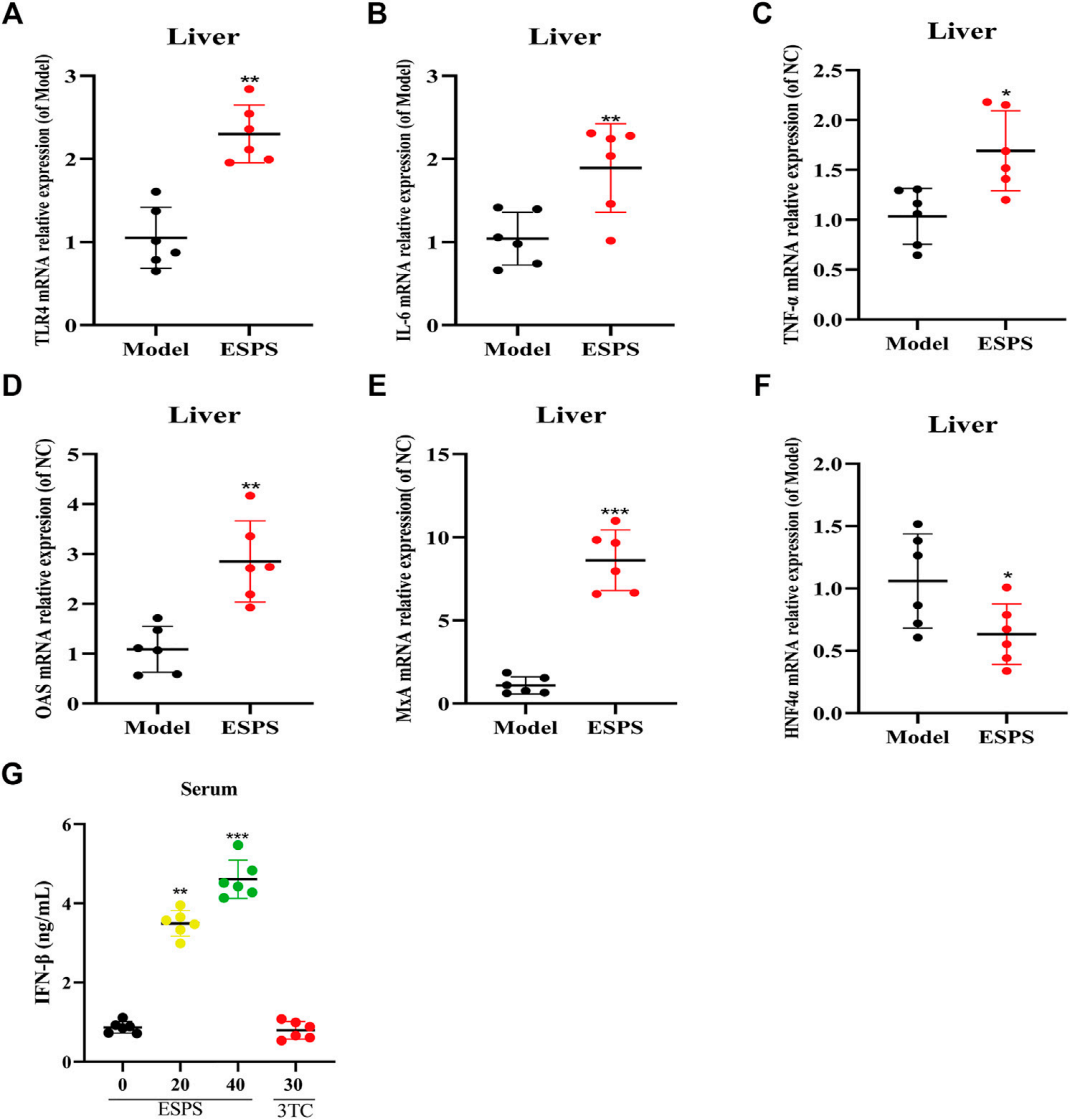
Influence on the innate immunomodulatory activity by ESPS *in vivo*. C57BL/6J mice were treated with ESPS (40 mg/kg/2 days) for 20 days. The mRNA relative expressions of TLR4 **(A)**, IL-6 **(B)**, TNF-α **(C)**, OAS **(D)**, MxA **(E)**, and HNF4α **(F)** in liver tissue of mice were detected by qPCR. Comparison between groups was performed by *t*-test analysis. Values are means ± SD (*n* = 6). *p < 0.05, **p < 0.01, ***p < 0.001 *vs*. the model group. Serum IFN-β was analyzed by ELISA kit **(G)**. Values are means ± SD (*n* = 6). ***p < 0.001 *vs*. the model group.

The results *in vitro* showed that ESPS played an anti-HBV role through innate immune signaling pathway mediated by TLR4. As expected, as shown in Figure 6, qPCR results showed that TLR4, OAS, MxA, IL-6, and TNF-α in liver tissue and IFN-β in serum were all upregulated in C57BL/6J HBV-tg mice treated with ESPS (40 mg/kg) for 20 days. It is suggested that polysaccharide ESPS may activate innate immunity and exert antiviral effect by TLR4. Notably, the downregulation of HNF4α in liver tissue implied that ESPS may inhibit HBV through reducing the activity of HBV core promotor.

## Discussion

In this study, we found that ESPS exhibited significant inhibitory activity on the replication of HBV *in vivo* and *in vitro*, and its antiviral activity has a time- and dose-dependent effect. The anti-HBV activity of ESPS may be associated with appropriate activation of TLR4 signaling pathway, which results in the enhancement of I interferon and pro-inflammatory factors. Therefore, the *Eupolyphaga sinensis* Walker–derived polysaccharide ESPS was valuable for further investigation as a novel innate immune response–associated anti-HBV agent.

Cell surface receptors play a vital role in identifying invading pathogens and initiating innate immune responses. For example, during HBV infection, both HBV DNA and pgRNA can be recognized by the cyclic GMP-AMP synthase (cGAS) and retinoic acid inducible gene I (RIG-I) receptors to activate the innate immune response (Marcellin et al., 2005; Megahed et al., 2020; Xie et al., 2020); however, HBV can still escape the innate immune response (Yu et al., 2017). ESPS, as a natural polysaccharide with immunostimulatory properties extracted from traditional Chinese medicine, can interact with cell surface receptors to activate the innate immune response to exert antiviral effects.

**FIGURE 7:**
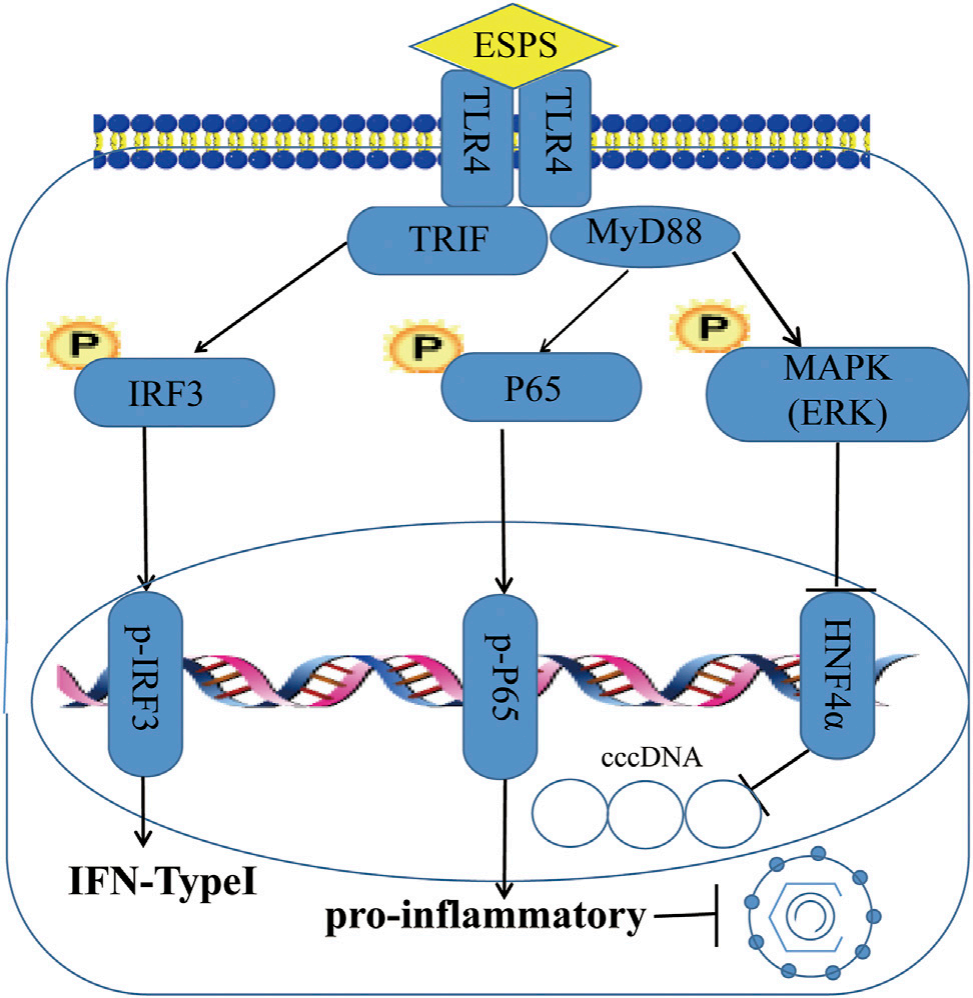
Schema of ESPS against HBV. ESPS promotes the expression of TLR4, thereby activating interferon signaling pathway and inflammatory signaling pathway to play an indirect antiviral role, or activating ERK pathway to directly inhibit HBV promoter activity and inhibit HBV replication.

As a nucleoside analogue, lamivudine targets the DNA polymerase of HBV, but its long-term application can cause mutations in the HBV-YMDD gene sequence, leading to HBV resistance (Marcellin et al., 2005). Unlike lamivudine, ESPS inhibited not only HBV DNA but also HBsAg, HBeAg, HBV RNA, and HBV core protein. Although the inhibitory effect of ESPS on intracellular HBV DNA was not as well as lamivudine, the inhibitory effect on the secretion of HBV DNA *in vitro* was comparable with lamivudine. Our data suggested that ESPS promoted the activation of cellular interferon system, stimulated the host to improve the level of interferon, and played an antiviral role. In addition, the *in vitro* data showed that ESPS inhibited HNF4α expression through the TLR4/NF-κB/ERK axis, thereby downregulating the activity of HBV core promoter, which may also be one of the mechanisms of ESPS for against HBV (Figure 7). Since monotherapy only exerts its antiviral effect on a single target site to varying degrees, and it has high probability to produce drug-resistant mutations, lamivudine combined with ESPS may produce better curative effect and break HBV resistance.

Polysaccharides are widely distributed in nature. A large number of experiments have proved that many biological polysaccharides have immune and anti-tumor effects (Xie et al., 2020). The previous research of our laboratory found that ESPS can stimulate immune cells, release cytokines, and improve the innate immune ability to fight tumors indirectly. Chronic HBV infection normally results in patients developing HCC, which suggests that ESPS may be able to restore or enhance innate immunity by activating immune cells and releasing cytokines to clear HBV and HBV-induced liver cancer.

In summary, we concluded that ESPS inhibited HBV replication *in vitro* and *in vivo via* activation of the TLR4 signaling pathway. Our study provided a reference for ESPS as an anti-HBV agent.

## Data Availability

The original contributions presented in the study are included in the article/Supplementary Material; further inquiries can be directed to the corresponding authors.

## Glossary

Amp: ampicillin
BSA: bovine serum albumin
CHB: chronic hepatitis B
cccDNA: covalently closed circular DNA
DAPI: diamidino-phenyl-indole
DMEM: Dulbecco’s modified Eagle’s medium
DMSO: dimethyl sulfoxide
ESPS: *Eupolyphaga sinensis* Walker polysaccharides
EDTA: ethylene diaminetetraacetic acid
FBS: fetal bovine serum
GFP: green fluorescent protein
HNF4a: hepatocyte nuclear factor 4 alpha
HBeAg: hepatitis B e antigen
HBsAg: hepatitis B surface antigen
HBV: hepatitis B virus
HBV pol: Hepatitis B virus pol protein
HBs: Hepatitis B virus S protein
HBc: Hepatitis B virus core protein
HBcAg: hepatitis B core antigen
HCC: hepatocellular carcinoma
HSPG: heparan sulfate proteoglycan
IF: immunofluorescence
IHC: immunohistochemistry
IL-6: interleukin-6
ISG: immune serum globulin
LPS: lipopolysaccharides
M: mol/L
NC: negative control
NK: natural killer cell
NTCP: cholic acid transporter-sodium taurocholate cotransporting polypeptide
OD: optical density
ORF: open reading frame
PBS: phosphate buffer saline
pgRNA: pregenomic RNA
PMSF: phenylmethanesulfonyl fluoride
qPCR: quantitative real-time PCR
rcDNA: relaxed circular DNA
SDS: sodium dodecyl sulfate
TBS: Tris–HCl buffer
TNF-a: tumor necrosis factor-α
3TC: lamivudine

## References

[B1] Belloni L. Allweiss L. Guerrieri F. Pediconi N. Volz T. Pollicino T. (2012). IFN-α Inhibits HBV Transcription and Replication in Cell Culture and in Humanized Mice by Targeting the Epigenetic Regulation of the Nuclear cccDNA Minichromosome. J. Clin. Invest. 122 (2), 529–537. 10.1172/JCI58847 22251702 PMC3266786

[B2] Carey I. Gersch J. Wang B. Moigboi C. Kuhns M. Cloherty G. (2020). Pregenomic HBV RNA and Hepatitis B Core-Related Antigen Predict Outcomes in Hepatitis B e Antigen-Negative Chronic Hepatitis B Patients Suppressed on Nucleos(T)ide Analogue Therapy. Hepatology 72 (1), 42–57. 10.1002/hep.31026 31701544

[B3] Chen L. Huang G. (2018). The Antiviral Activity of Polysaccharides and Their Derivatives. Int. J. Biol. Macromol 115, 77–82. 10.1016/j.ijbiomac.2018.04.056 29654857

[B4] Fang R. Xiao Y. Zhong W. H. Vincent K. W. W. Hong Y. X. Ji H. R. (2019). 6-Aminonicotinamide, a Novel Inhibitor of Hepatitis B Virus Replication and HBsAg Production. EBioMedicine 49, 232–246. 10.1016/j.ebiom.2019.10.022 31680002 PMC6945246

[B5] Higashi-Kuwata N. Hayashi S. Kumamoto H. Ogata-Aoki H. Das D. Venzon D. (2021). Identification of a Novel Long-Acting 4'-modified Nucleoside Reverse Transcriptase Inhibitor against HBV. J. Hepatol. 74 (5), 1075–1086. 10.1016/j.jhep.2020.12.006 33333207 PMC9703152

[B6] Kawai T. Akira S. (2011). Toll-like Receptors and Their Crosstalk with Other Innate Receptors in Infection and Immunity. Immunity 34, 637–650. 10.1016/j.immuni.2011.05.006 21616434

[B7] Li L. Li Y. Xiong Z. Shu W. Yang Y. Guo Z. (2019). FoxO4 Inhibits HBV Core Promoter Activity through ERK-Mediated Downregulation of HNF4α. Antivir. Res 170, 104568. 10.1016/j.antiviral.2019.104568 31351930

[B8] Likhitsup A. Razumilava N. Parikh N. D. (2019). Treatment for Advanced Hepatocellular Carcinoma: Current Standard and the Future. Clin. Liver Dis. (Hoboken) 13, 13–19. 10.1002/cld.782 31168360 PMC6465790

[B9] Lin Z. Liao W. Ren J. (2016). Physicochemical Characterization of a Polysaccharide Fraction from Platycladus Orientalis (L.) Franco and its Macrophage Immunomodulatory and Anti-hepatitis B Virus Activities. J. Agric. Food Chem. 64 (29), 5813–5823. 10.1021/acs.jafc.6b01387 27345527

[B10] Long X. Zhang Z. Han S. Tang M. Zhou J. Zhang J. (2015). Structural Mediation on Polycation Nanoparticles by Sulfadiazine to Enhance DNA Transfection Efficiency and Reduce Toxicity. ACS Appl. Mater. Inter. 7 (14), 7542–7551. 10.1021/am508847j 25801088

[B11] Lopatin U. (2019). Drugs in the Pipeline for HBV. Clin. Liver Dis. 23, 535–555. 10.1016/j.cld.2019.04.006 31266626

[B12] Luangsay S. Gruffaz M. Isorce N. Testoni B. Michelet M. Faure-Dupuy S. (2015). Early Inhibition of Hepatocyte Innate Responses by Hepatitis B Virus. J. Hepatol. 63 (6), 1314–1322. 10.1016/j.jhep.2015.07.014 26216533

[B13] Marcellin P. Asselah T. Boyer N. (2005). Treatment of Chronic Hepatitis B J Viral. Hepat 12 (4), 333–345. 10.1111/j.1365-2893.2005.00599.x 15985003

[B14] Megahed F. A. K. Zhou X. Sun P. (2020). The Interactions between HBV and the Innate Immunity of Hepatocytes. Viruses 12 (3), 285. 10.3390/v12030285 PMC7150781 32151000

[B15] Namineni S. O'Connor T. Faure-Dupuy S. Johansen P. Riedl T. Liu K. (2020). A Dual Role for Hepatocyte-Intrinsic Canonical NF-κB Signaling in virus Control. J. Hepatol. 72 (5), 960–975. 10.1016/j.jhep.2019.12.019 31954207

[B16] Niu C. Li L. Daffis S. Lucifora J. Bonnin M. Maadadi S. (2018). Toll-like Receptor 7 Agonist GS-9620 Induces Prolonged Inhibition of HBV via a Type I Interferon-dependent Mechanism. J. Hepatol. 68 (5), 922–931. 10.1016/j.jhep.2017.12.007 29247725

[B17] Revill P. Testoni B. Locarnini S. Zoulim F. (2016). Global Strategies Are Required to Cure and Eliminate HBV Infection. Nat. Rev. Gastroenterol. Hepatol. 13 (4), 239–248. 10.1038/nrgastro.2016.7 26907881

[B18] Takeuchi O. Akira S. (2010). Pattern Recognition Receptors and Inflammation. Cell 140, 805–820. 10.1016/j.cell.2010.01.022 20303872

[B19] Tang Q. F. Dai Y. Liu X. L. (2010). Immunomodulatory Effects of Orally Administered Aqueous Extract from Eupolyphaga Sinensis Walker. AFR. J. Biotechnol. 9 (50), 8682–8686.

[B20] Vittal A. Ghany M. G. (2019). WHO Guidelines for Prevention, Care and Treatment of Individuals Infected with HBV: a US Perspective. Clin. Liver Dis. 23, 417–432. 10.1016/j.cld.2019.04.008 31266617 PMC9616205

[B21] Wu J. Huang S. Zhao X. Chen M. Lin Y. Xia Y. (2014). Poly(I:C) Treatment Leads to Interferon-dependent Clearance of Hepatitis B Virus in a Hydrodynamic Injection Mouse Model. J. Virol. 88 (18), 10421–10431. 10.1128/JVI.00996-14 24920792 PMC4178898

[B22] Wu J. Meng Z. Jiang M. Pei R. Trippler M. Broering R. (2009). Hepatitis B Virus Suppresses Toll-like Receptor-Mediated Innate Immune Responses in Murine Parenchymal and Nonparenchymal Liver Cells. Hepatology 49 (4), 1132–1140. 10.1002/hep.22751 19140219

[B23] Wu J. Zhao Y. Park Y. K. Lee J. Y. Gao L. Zhao J. (2018). Loss of PDK4 Switches the Hepatic NF-Κb/TNF Pathway from Pro-survival to Pro-apoptosis. Hepatology 68 (3), 1111–1124. 10.1002/hep.29902 29603325 PMC6165716

[B24] Wu W. Ren Q. Li C. Wang Y. Sang M. Zhang Y. (2013). Characterization and Comparative Profiling of MicroRNAs in a Sexual Dimorphism Insect, Eupolyphaga Sinensis Walker. PLoS ONE 8, e59016. 10.1371/journal.pone.0059016 23620723 PMC3631196

[B25] Xie X. Shen W. Zhou Y. Ma L. Xu D. Ding J. (2020). Characterization of a Polysaccharide from Eupolyphaga Sinensis walker and its Effective Antitumor Activity via Lymphocyte Activation. Int. J. Biol. Macromol 162, 31–42. 10.1016/j.ijbiomac.2020.06.120 32553956

[B26] Yang H. Zhou Z. He L. Ma H. Qu W. Yin J. (2018). Hepatoprotective and Inhibiting HBV Effects of Polysaccharides from Roots of Sophora Flavescens. Int. J. Biol. Macromol 108, 744–752. 10.1016/j.ijbiomac.2017.10.171 29111266

[B27] Yu X. Lan P. Hou X. Han Q. Lu N. Li T. (2017). HBV Inhibits LPS-Induced NLRP3 Inflammasome Activation and IL-1β Production via Suppressing the NF-Κb Pathway and ROS Production. J. Hepatol. 66 (4), 693–702. 10.1016/j.jhep.2016.12.018 28027970

[B28] Yuen M. F. Chen D. S. Dusheiko G. M. Janssen H. L. A. Lau D. T. Y. Locarnini S. A. (2018). Hepatitis B Virus Infection. Nat. Rev. Dis. Primers 4, 18035. 10.1038/nrdp.2018.35 29877316

[B29] Zeisel M. B. Lucifora J. Mason W. S. Sureau C. Beck J. Levrero M. (2015). Towards an HBV Cure: State-Of-The-Art and Unresolved Questions–Report of the ANRS Workshop on HBV Cure. Gut 64, 1314–1326. 25670809 10.1136/gutjnl-2014-308943

[B30] Zhan Y. Zhang H. Liu R. Wang W. Qi J. Zhang Y. (2016). Eupolyphaga Sinensis Walker Ethanol Extract Suppresses Cell Growth and Invasion in Human Breast Cancer Cells. Integr. Cancer Ther. 15 (1), 102–112. 10.1177/1534735415598224 26242891 PMC5736081

[B31] Zhang M. Wu X. Lai F. Zhang X. Wu H. Min T. (2016). Betaine Inhibits Hepatitis B Virus with an Advantage of Decreasing Resistance to Lamivudine and Interferon α. J. Agric. Food Chem. 64 (20), 4068–4077. 10.1021/acs.jafc.6b01180 27144395

[B32] Zoulim F. Lebossé F. Levrero M. (2016). Current Treatments for Chronic Hepatitis B Virus Infections. Curr. Opin. Virol. 18, 109–116. 10.1016/j.coviro.2016.06.004 27318098

