# Supplementary material for: A Polysaccharide From Eupolyphaga sinensis Walker With Anti-HBV Activities In Vitro and In Vivo
Source: Front Pharmacol. 2022 Mar 3;13:827128. doi: 10.3389/fphar.2022.827128 (PMC8928433; doi:10.3389/fphar.2022.827128)
Supplement: Supplementary file 1 [file DataSheet1.PDF]

Figure 1G

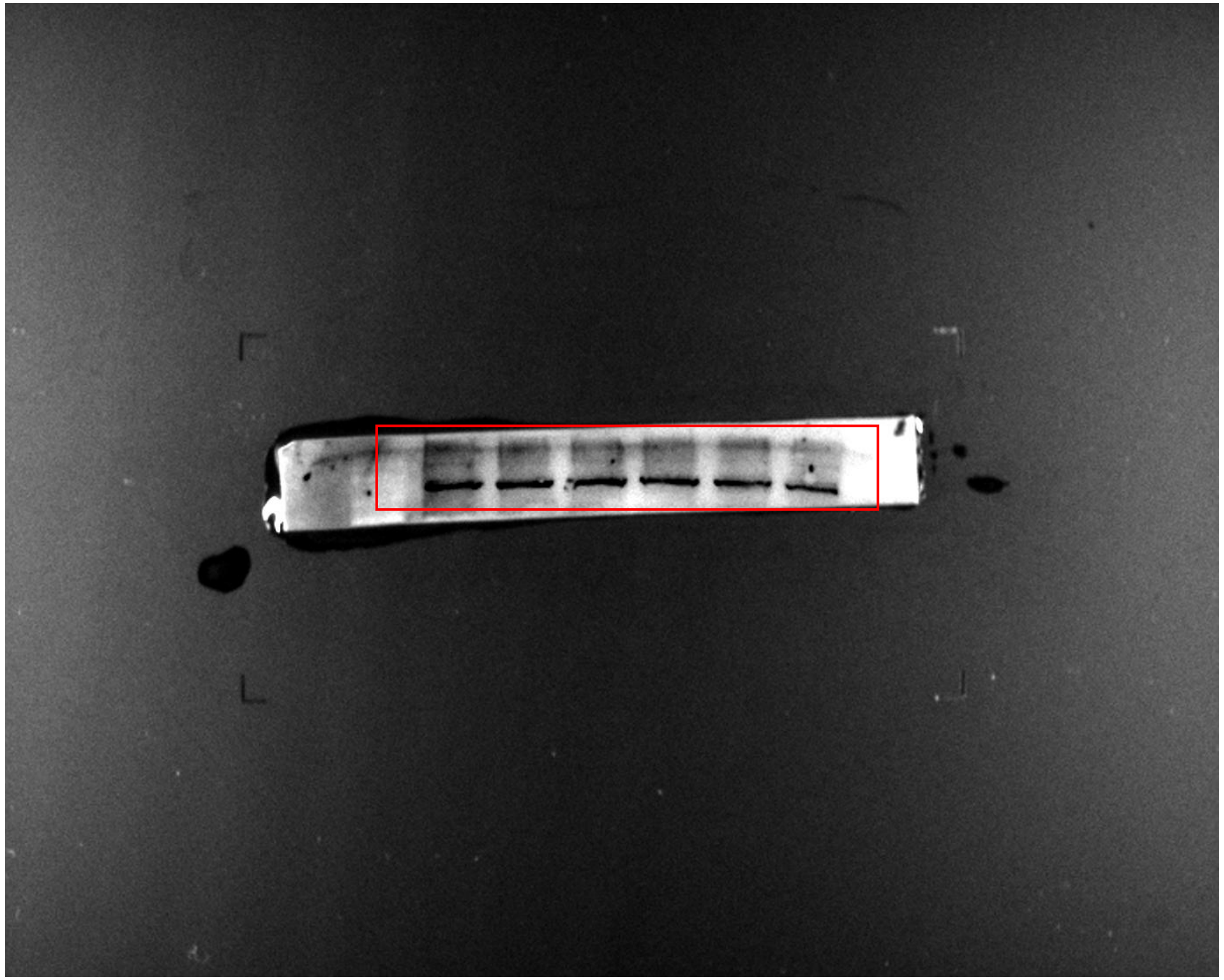

Figure 1G

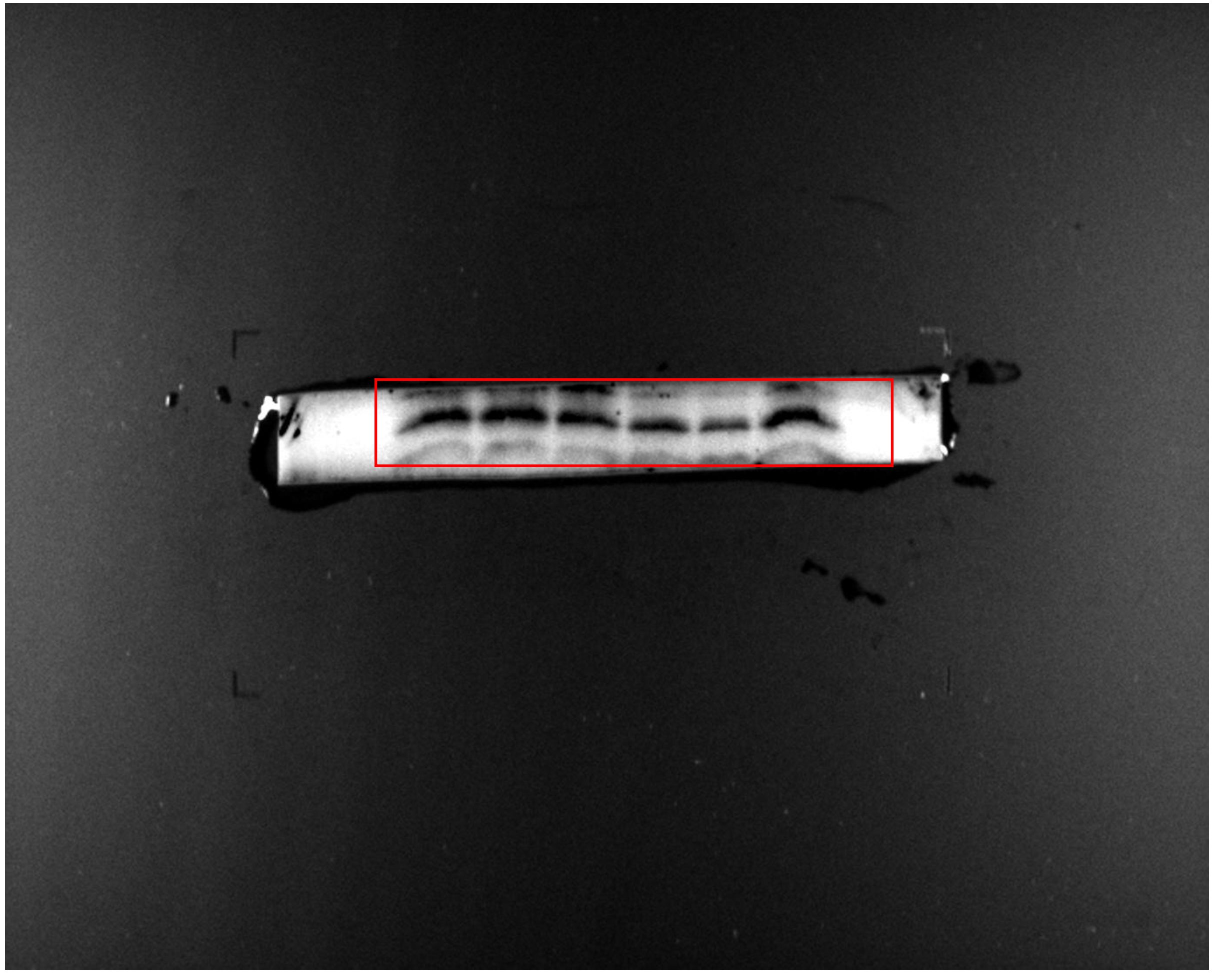

Figure 1G

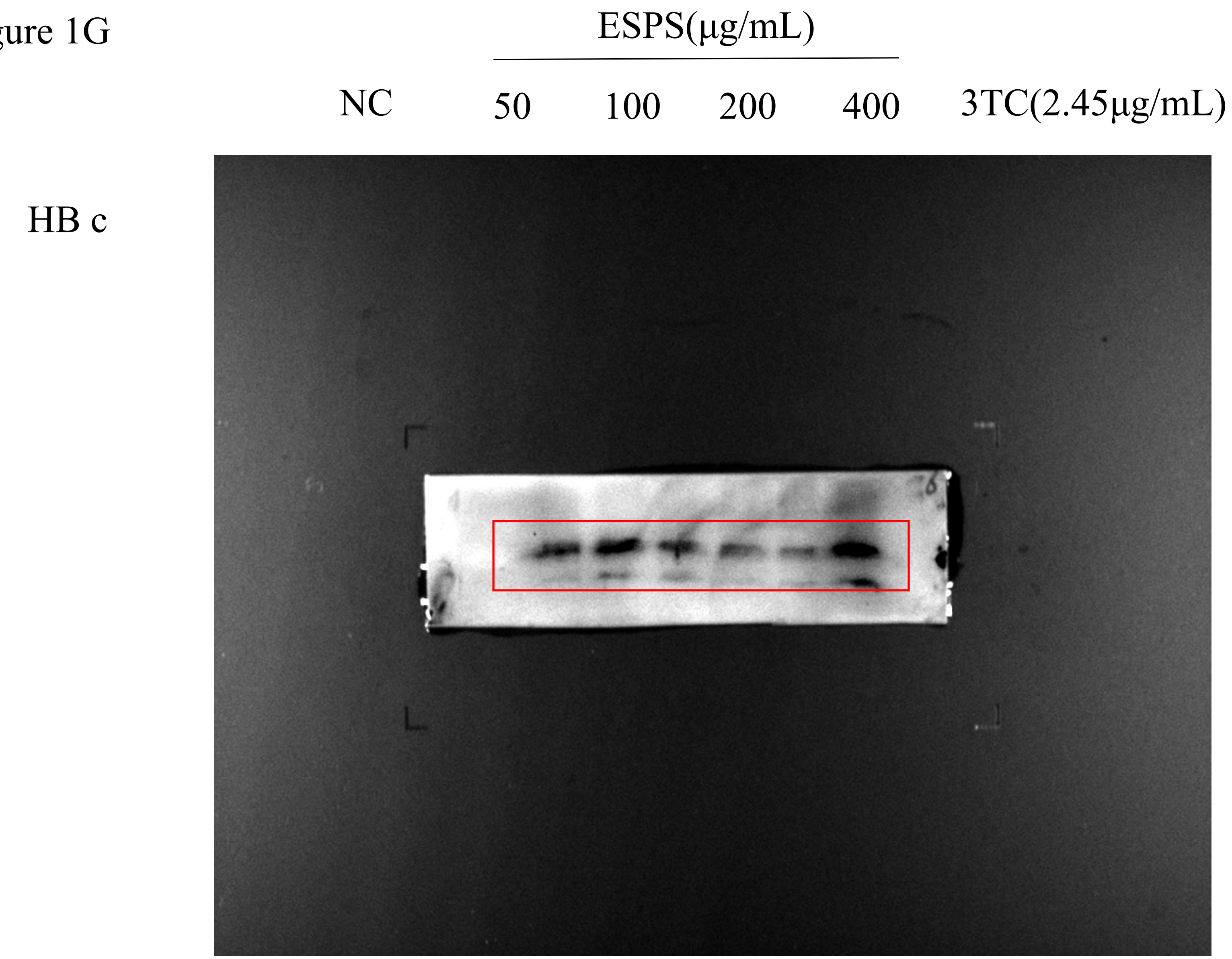

Figure 1G

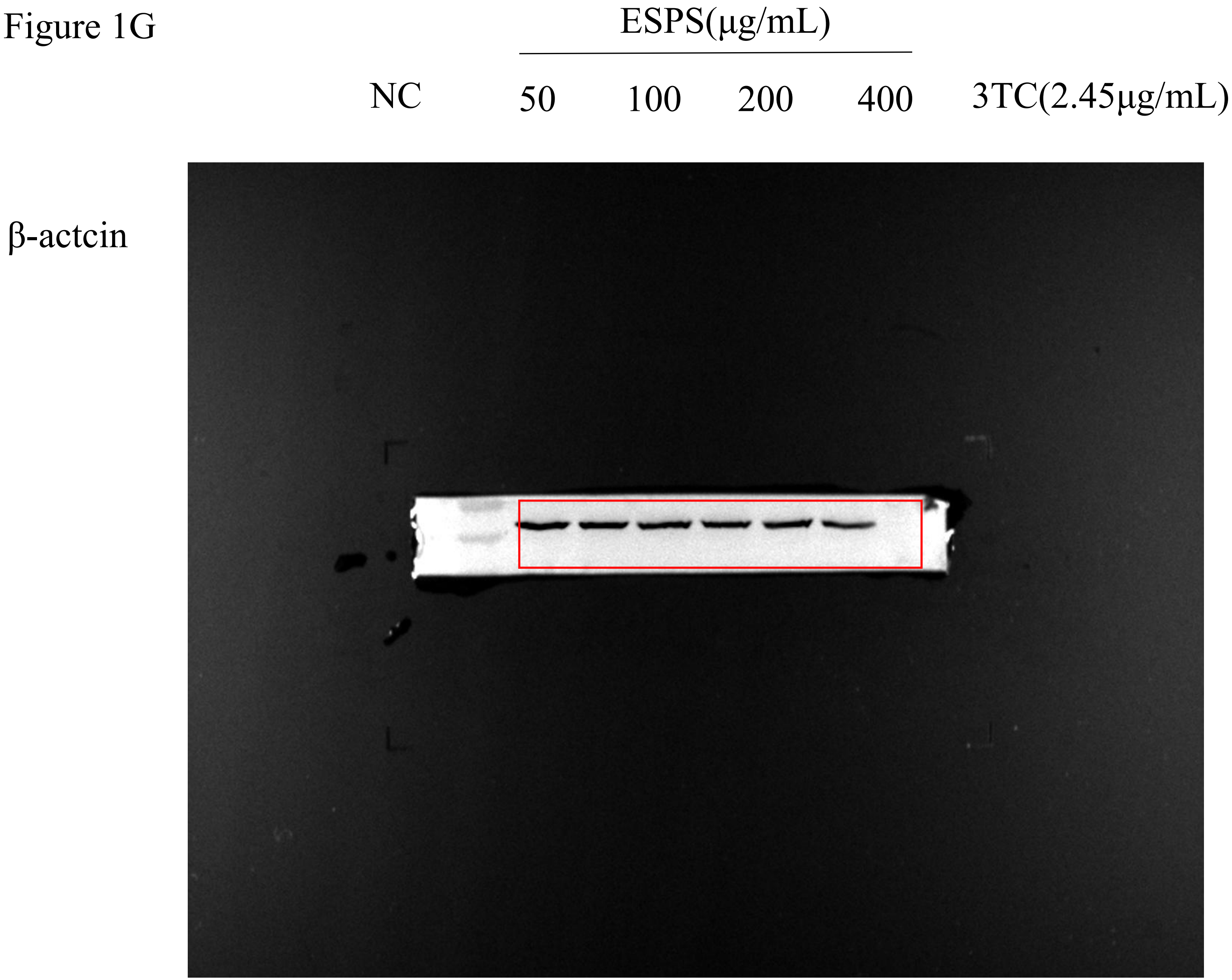

Figure 2A

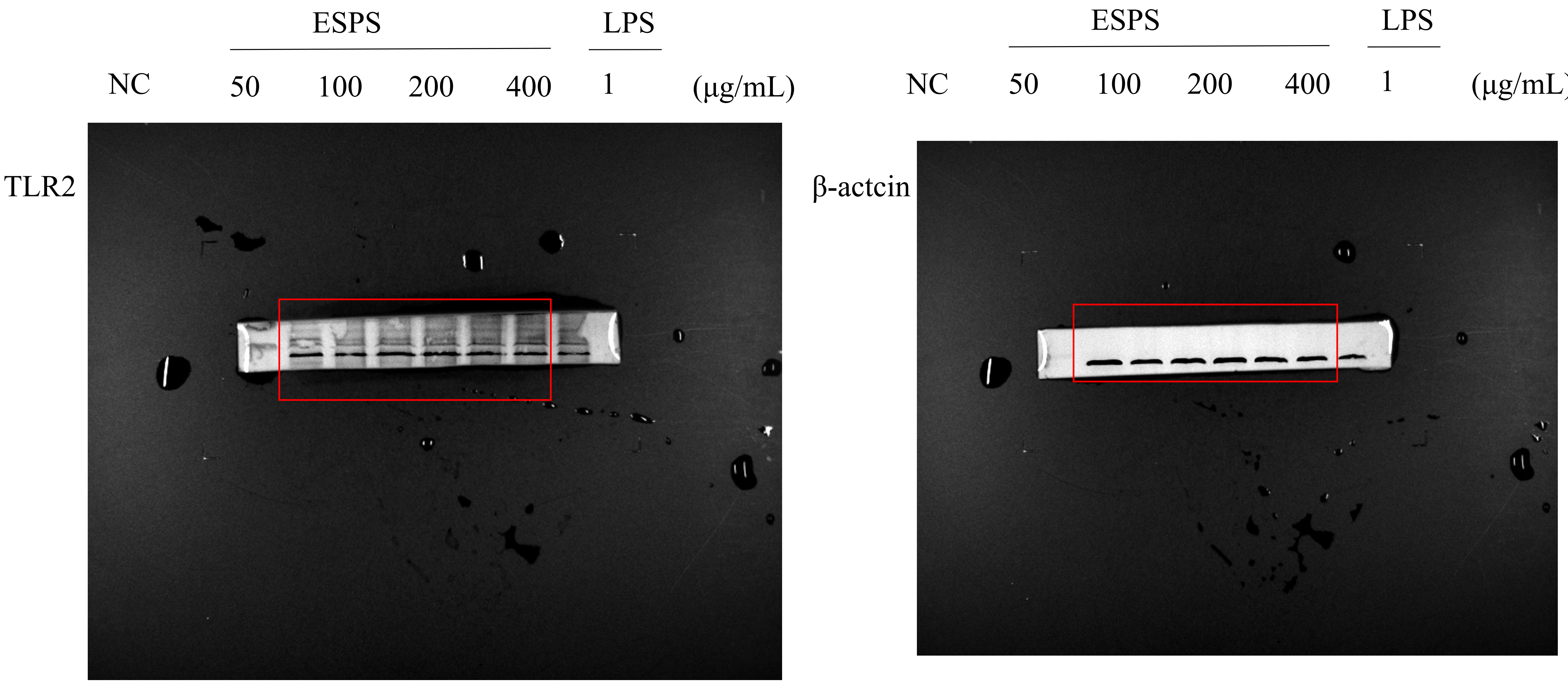

The seventh lane is another polysaccharide named SEP， which was extracted in our lab . Its antiviral effect has been reported and can be used as a positive control, but it is not discussed in this article, so we cut it off.

Figure 2C

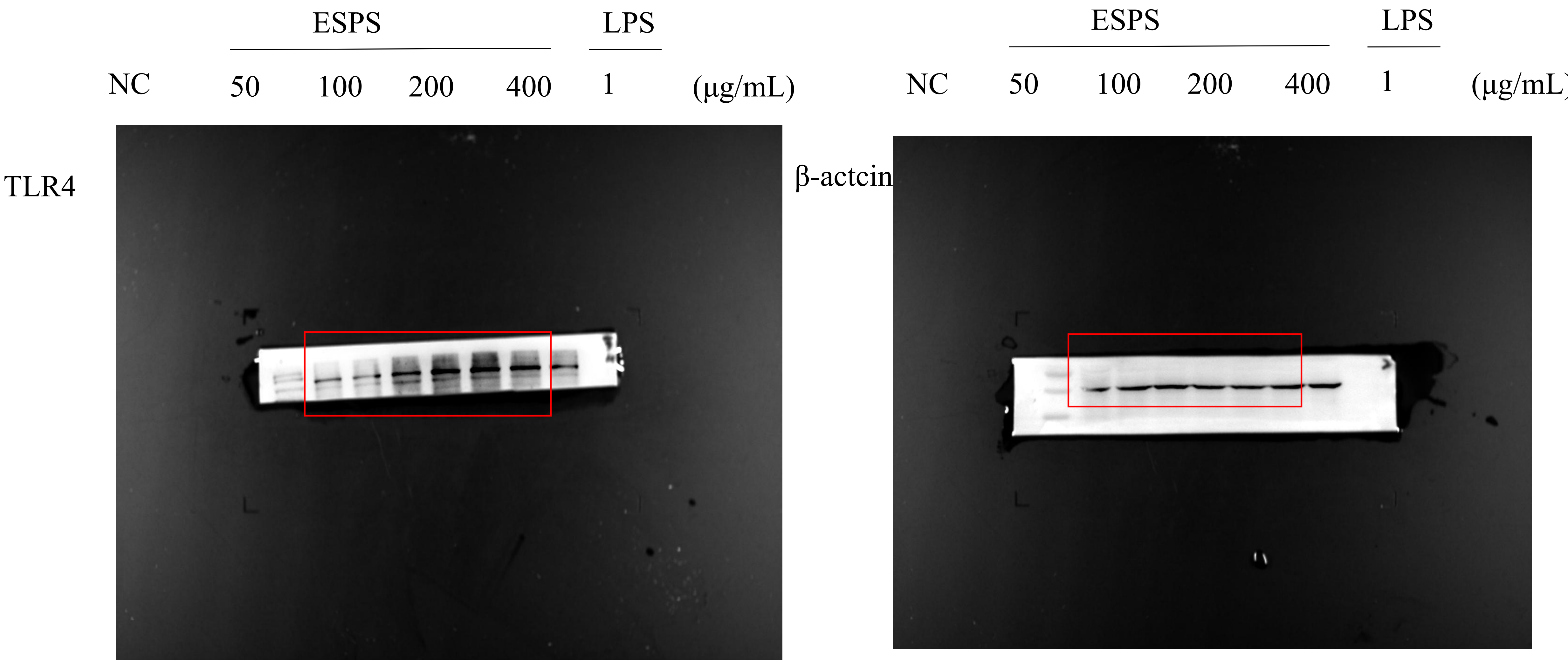

The seventh lane is another polysaccharide named SEP， which was extracted in our lab . Its antiviral effect has been reported and can be used as a positive control, but it is not discussed in this article, so we cut it off.

Figure 3A

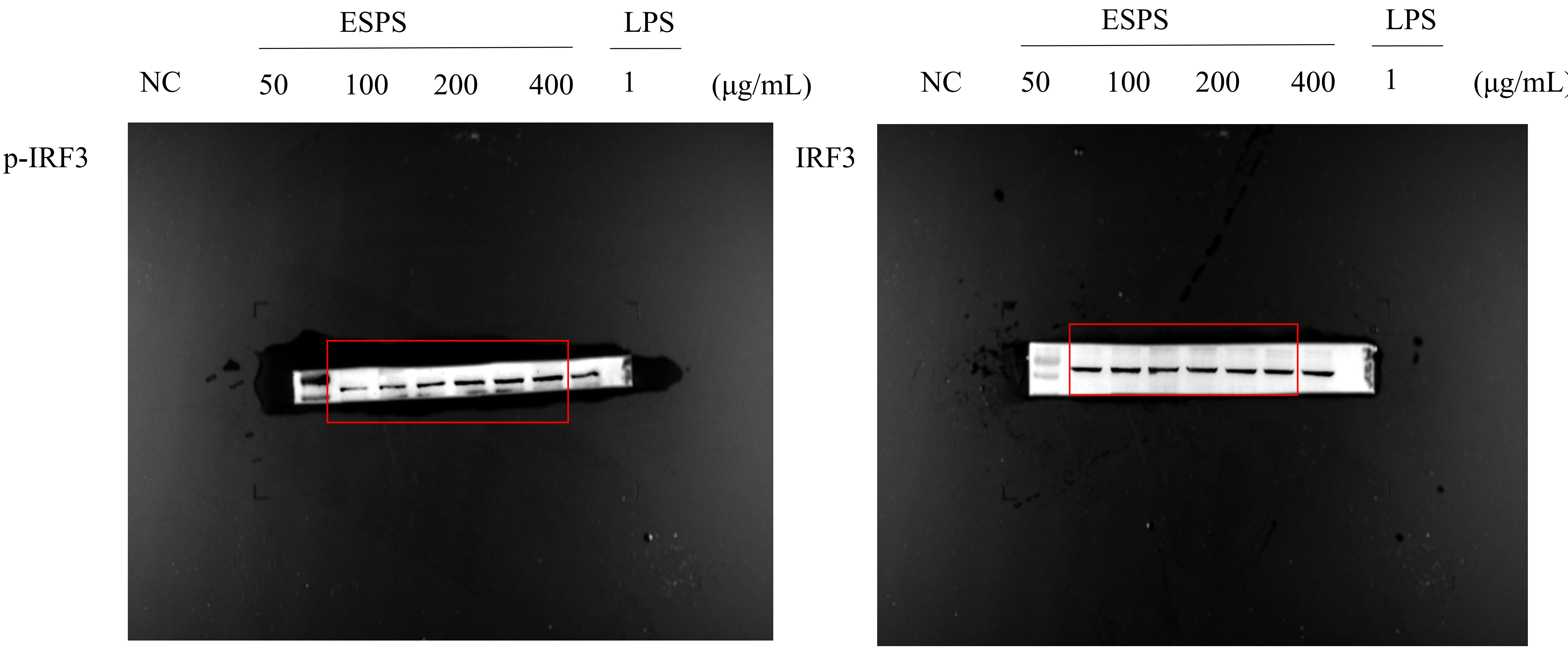

The seventh lane is another polysaccharide named SEP， which was extracted in our lab . Its antiviral effect has been reported and can be used as a positive control, but it is not discussed in this article, so we cut it off.

Figure 3A

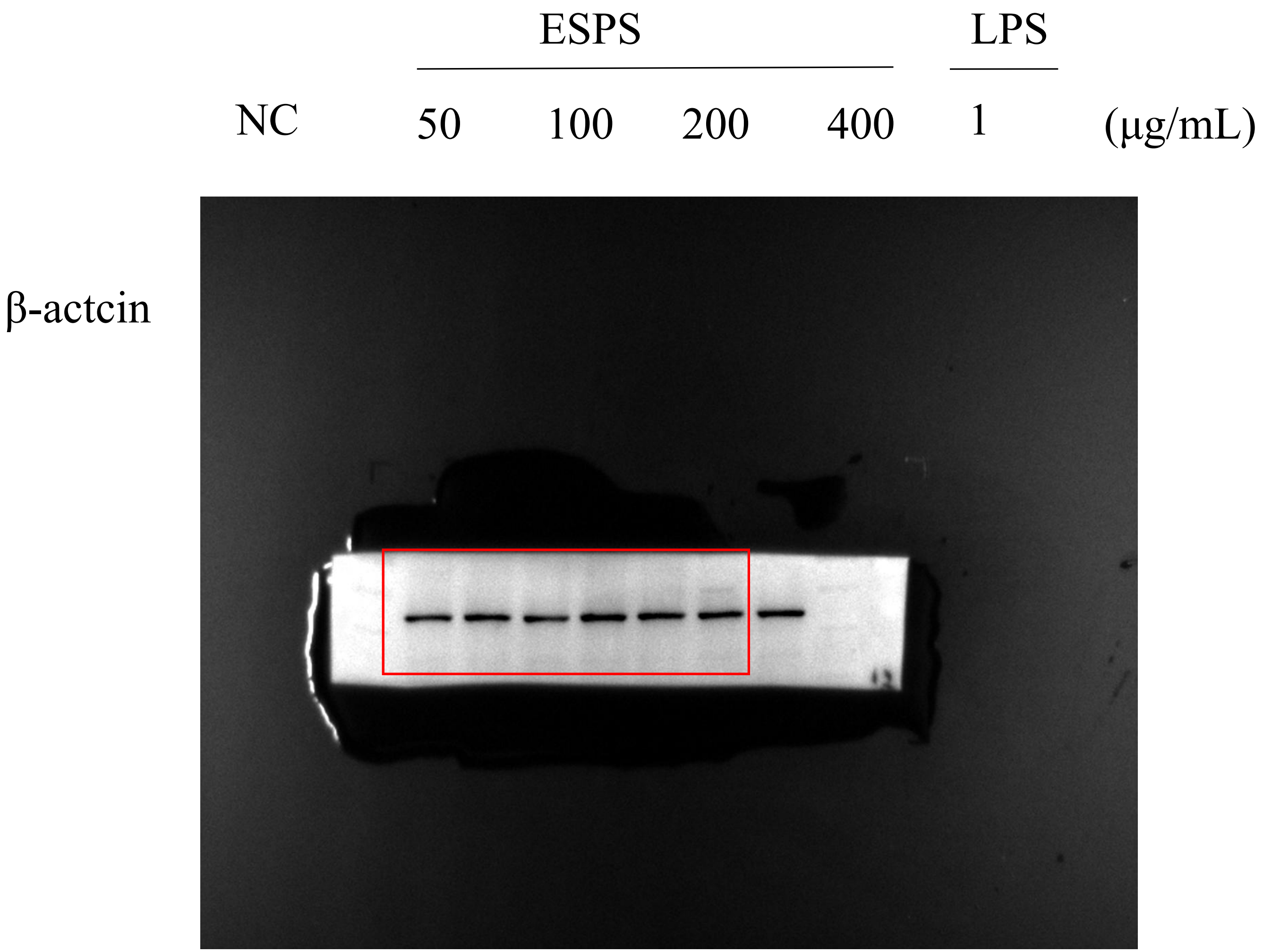

The seventh lane is another polysaccharide named SEP， which was extracted in our lab . Its antiviral effect has been reported and can be used as a positive control, but it is not discussed in this article, so we cut it off.

Figure 4A

ESPS LPS

NC 50 100 200 400 1 (μg/mL)

p-P65

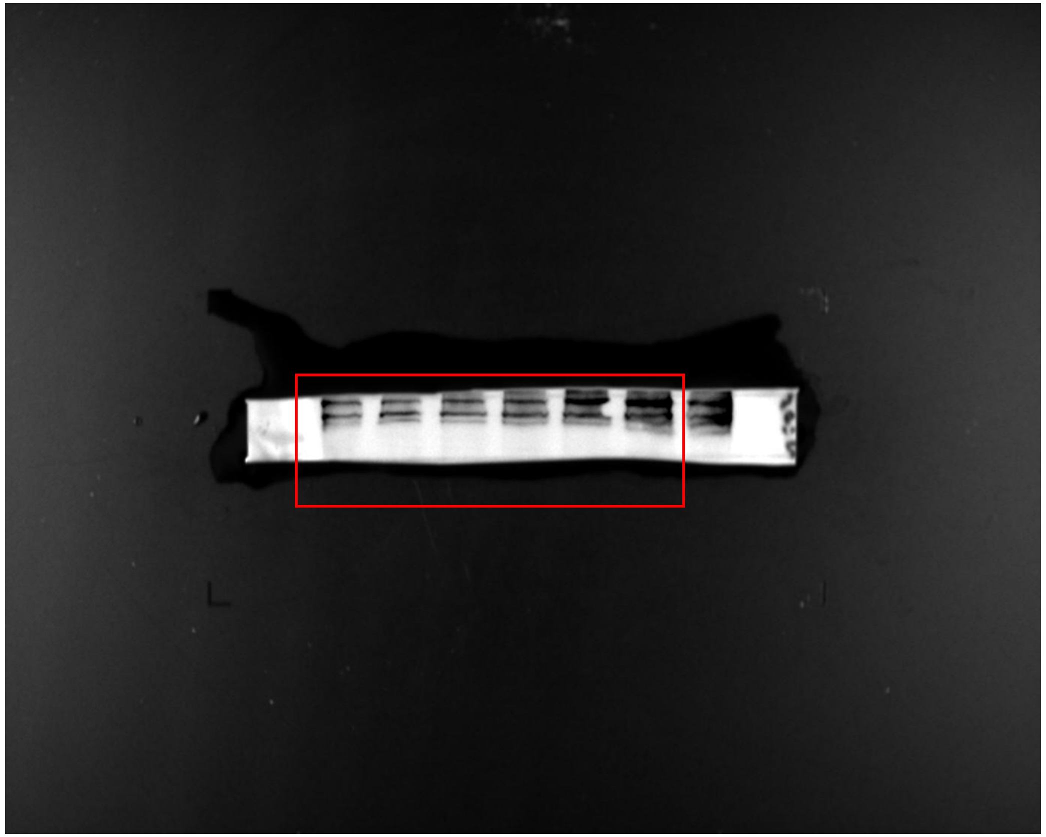

ESPS LPS

NC 50 100 200 400 1 (μg/mL)

P65

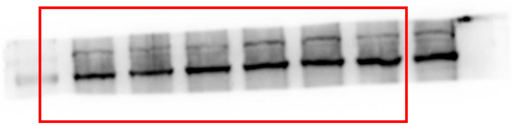

The seventh lane is another polysaccharide named SEP， which was extracted in our lab . Its antiviral effect has been reported and can be used as a positive control, but it is not discussed in this article, so we cut it off.

Figure 4A

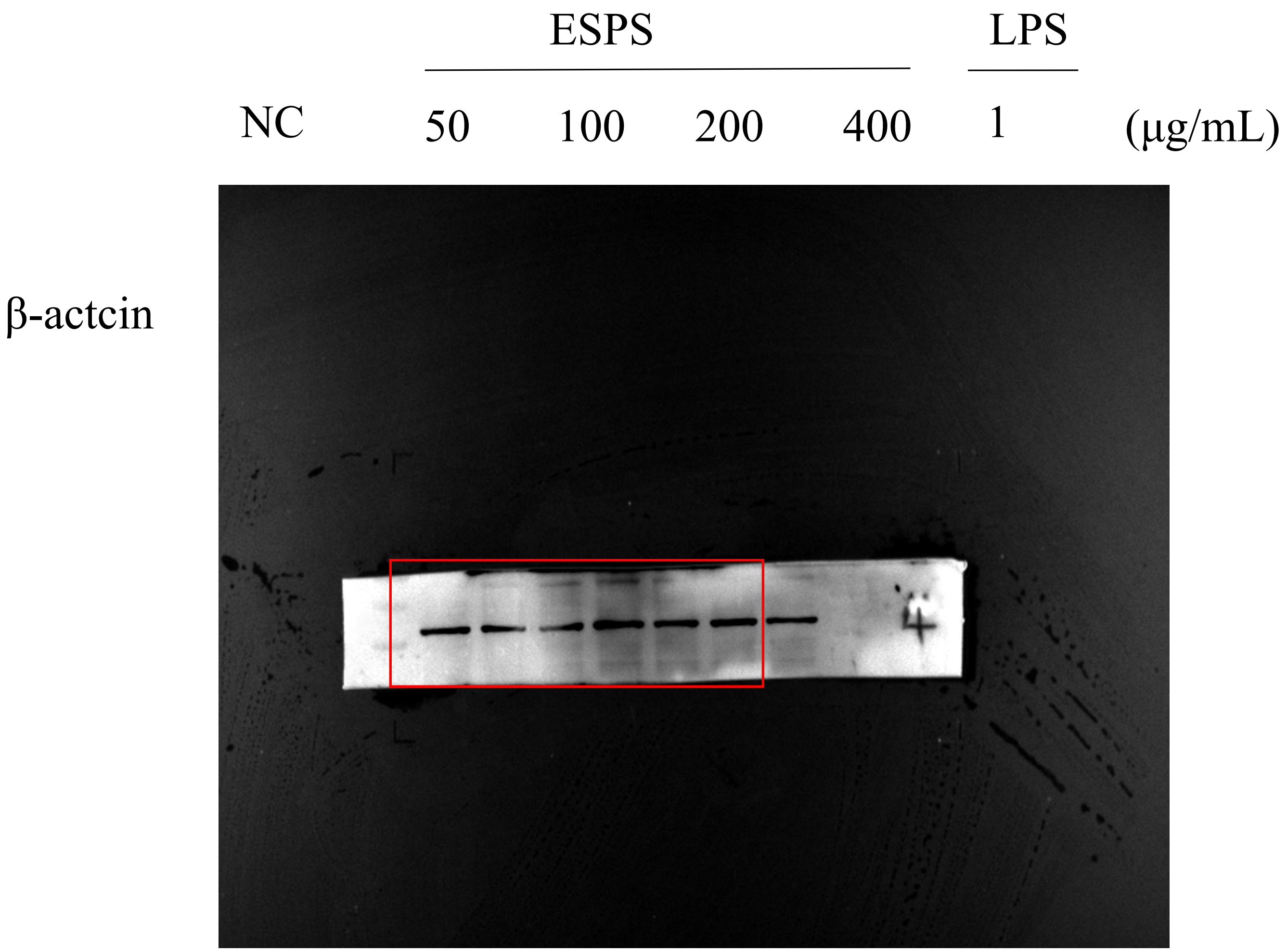

The seventh lane is another polysaccharide named SEP， which was extracted in our lab . Its antiviral effect has been reported and can be used as a positive control, but it is not discussed in this article, so we cut it off.

Figure 4E

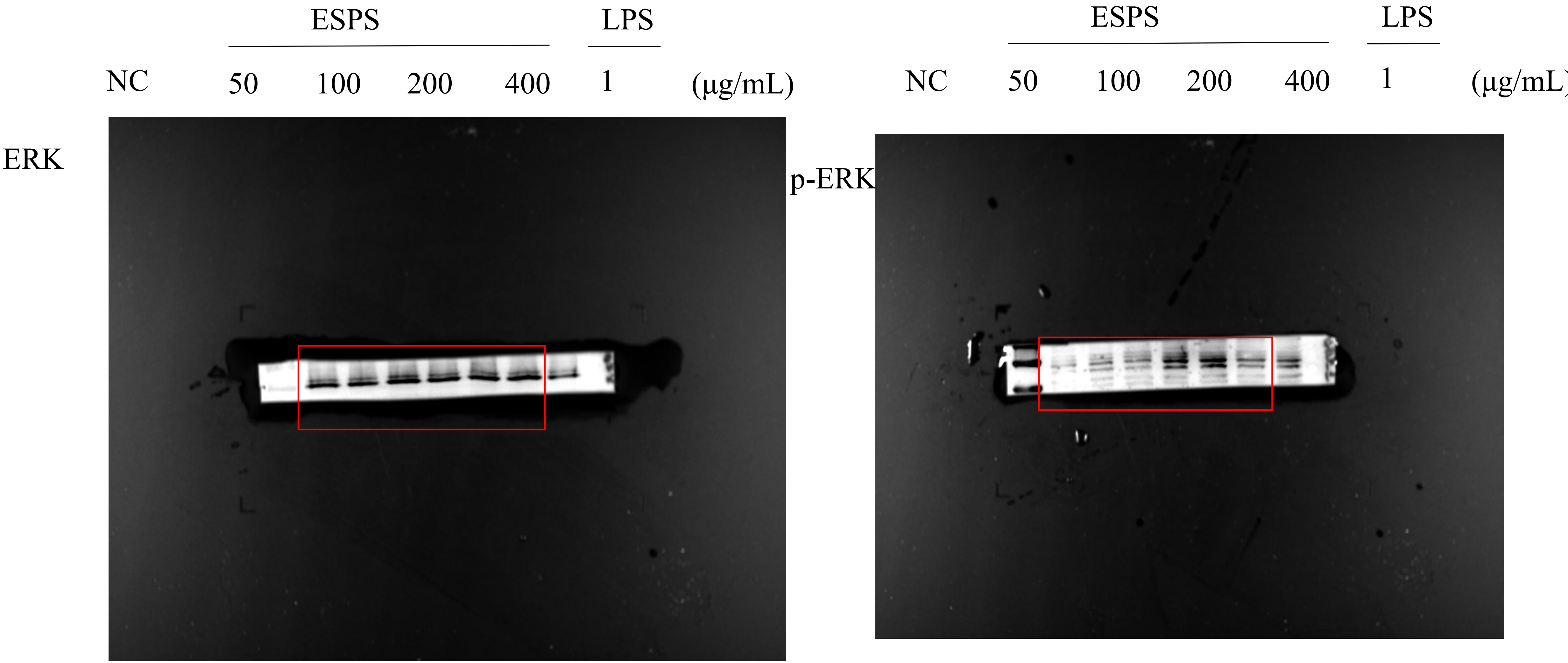

The seventh lane is another polysaccharide named SEP， which was extracted in our lab . Its antiviral effect has been reported and can be used as a positive control, but it is not discussed in this article, so we cut it off.

Figure 4E

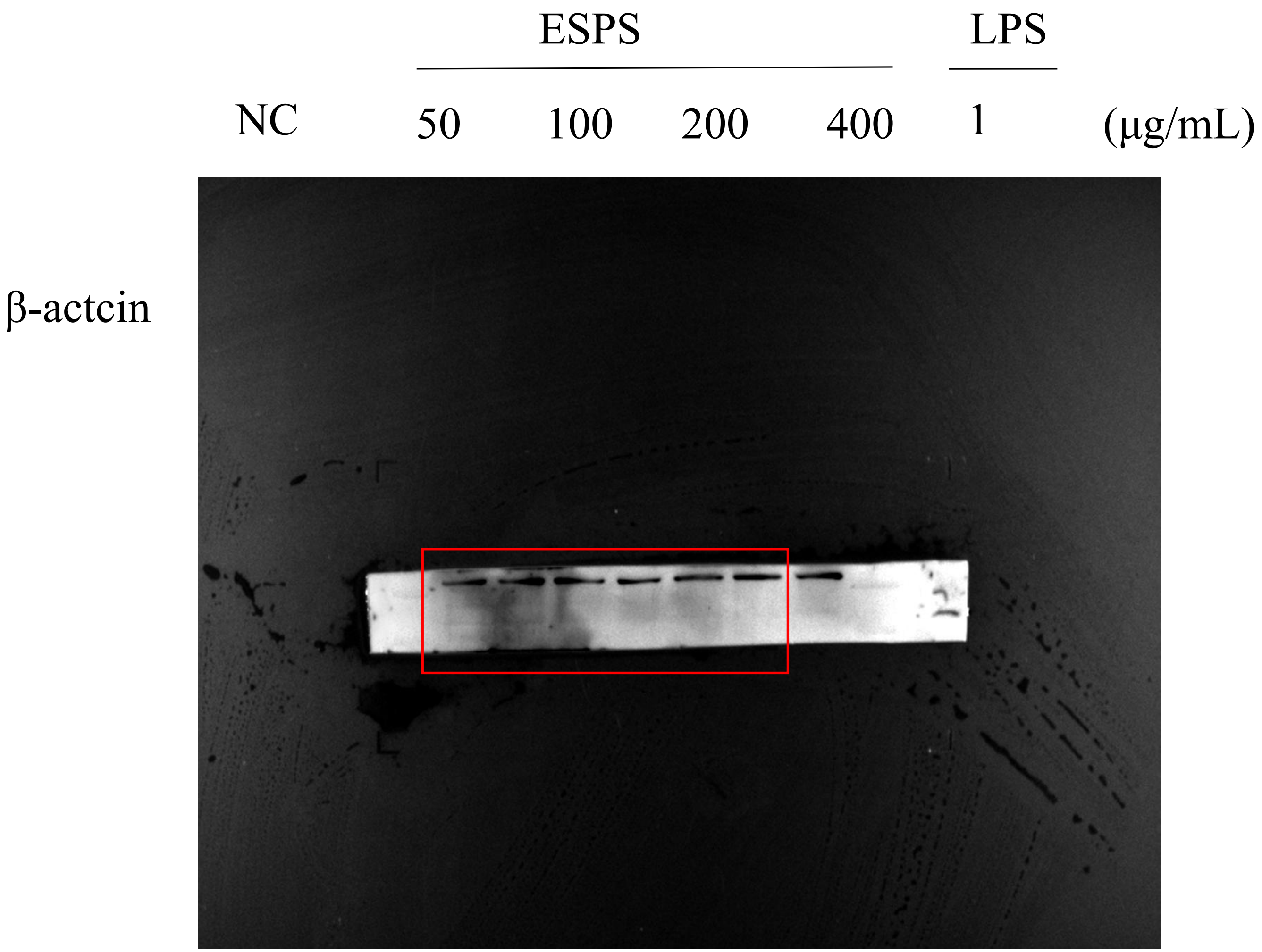

The seventh lane is another polysaccharide named SEP， which was extracted in our lab . Its antiviral effect has been reported and can be used as a positive control, but it is not discussed in this article, so we cut it off.

Figure 4G HepG2.2.15

ESPS

NC 50 100 200 400 (μg/mL)

HNF-4α

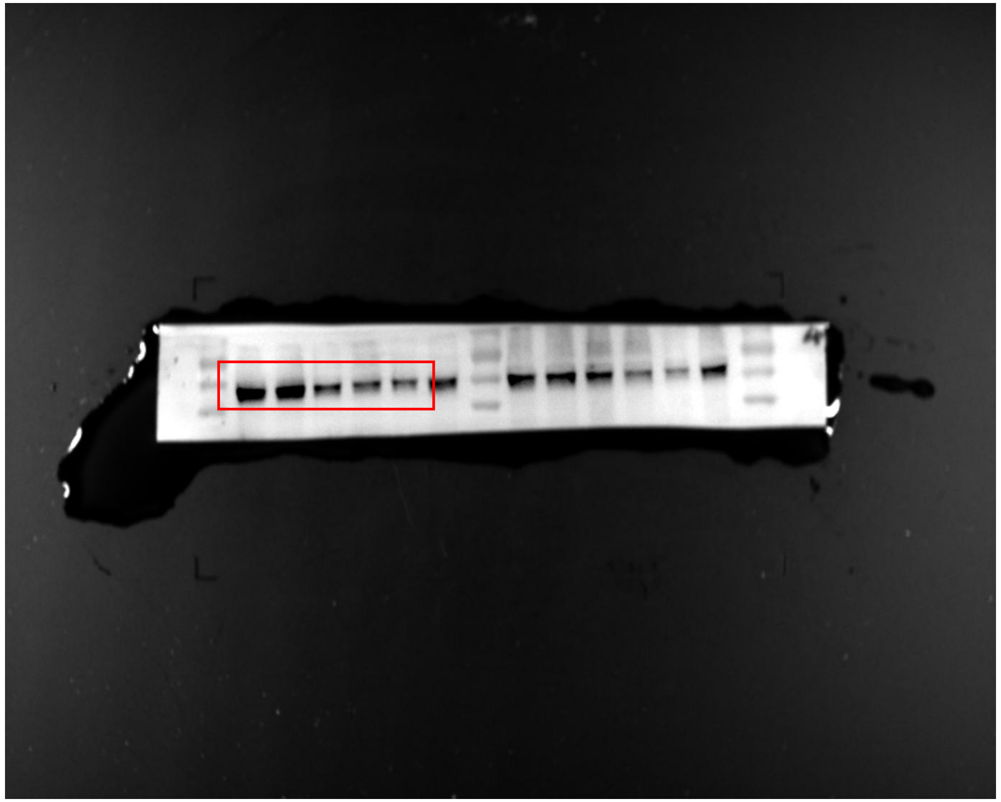

ESPS

NC 50 100 200 400 (μg/mL)

β-actcin

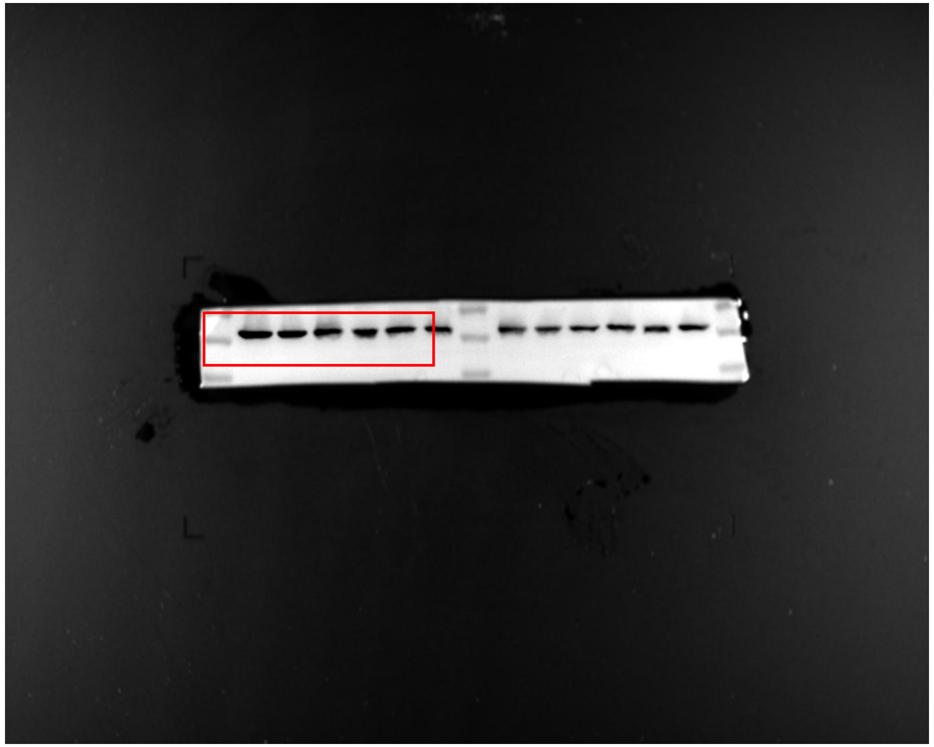

The seventh lane is another polysaccharide named SEP， which was extracted in our lab . Its antiviral effect has been reported and can be used as a positive control, but it is not discussed in this article, so we cut it off.

Figure 4G Huh 7

ESPS

NC 50 100 200 400 (μg/mL)

HNF-4α

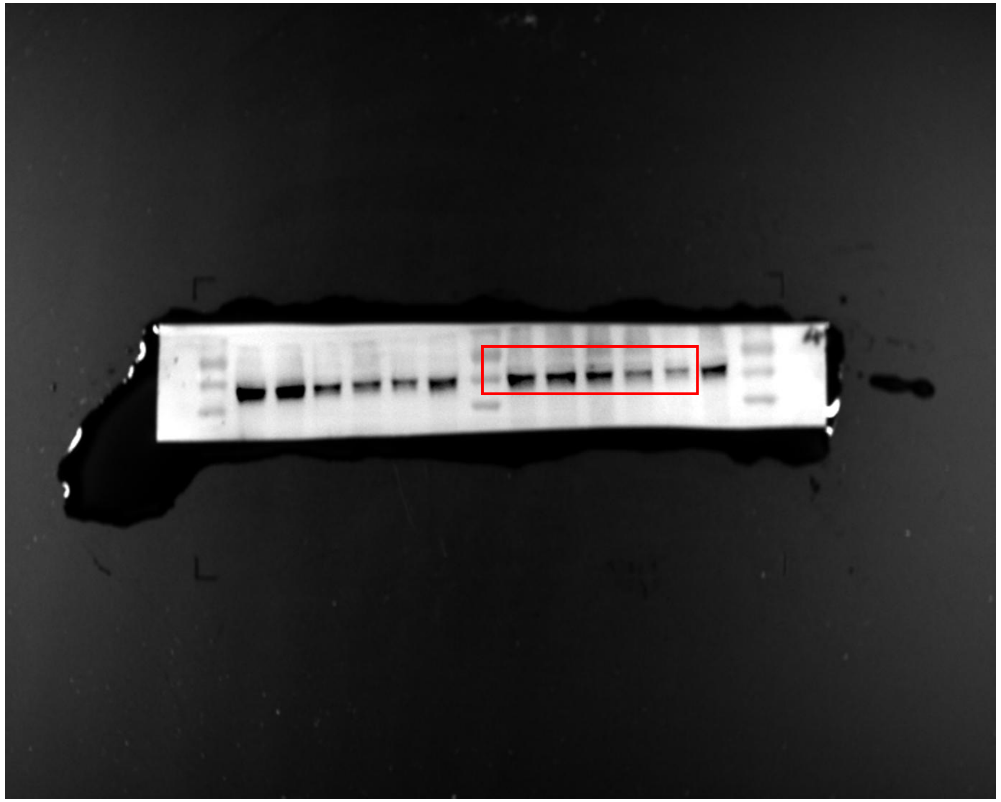

β-actcin

ESPS

NC 50 100 200 400 (μg/mL)

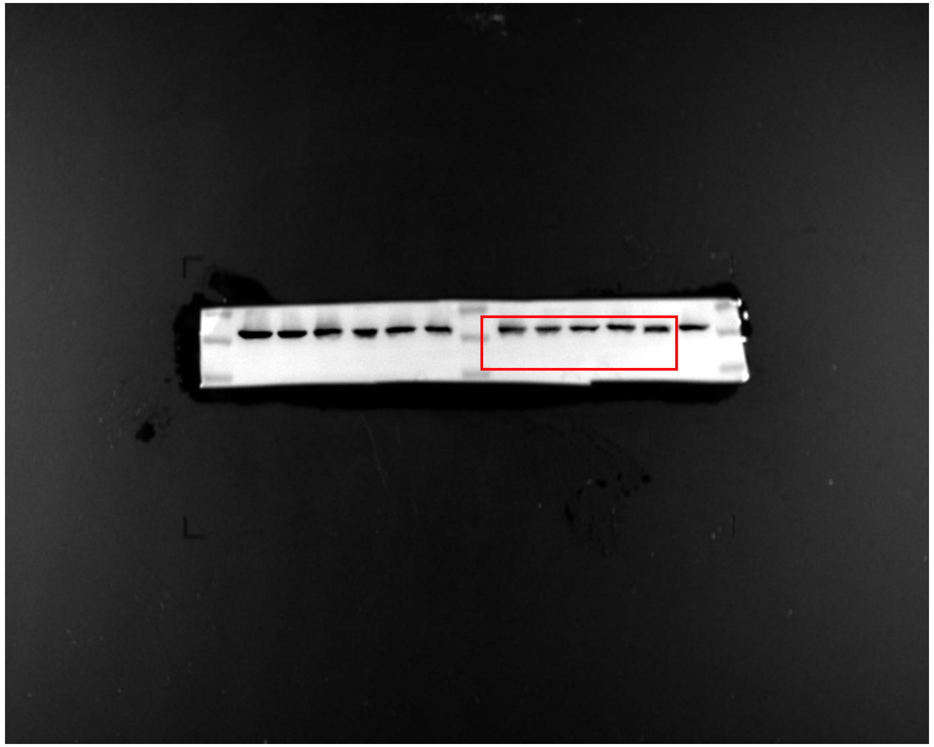

The sixth lane and twelfth lane are another polysaccharide named SEP， which was extracted in our lab . Its antiviral effect has been reported and can be used as a positive control, but it is not discussed in this article, so we cut it off.
