# Supplementary material for: A Polysaccharide From Eupolyphaga sinensis Walker With Anti-HBV Activities In Vitro and In Vivo
Source: Front Pharmacol. 2022 Mar 3;13:827128. doi: 10.3389/fphar.2022.827128 (PMC8928433; doi:10.3389/fphar.2022.827128)
Supplement: Supplementary file 2 [file Image1.pdf]

Supplementary Material

1.1 Supplementary Figures

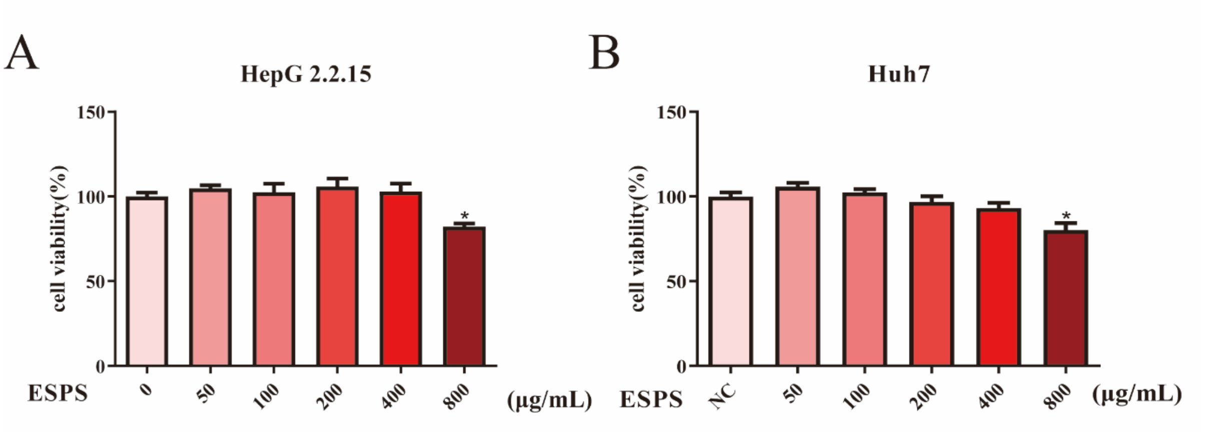

Supplementary Figure 1. The cytotoxicity assay of ESPS on hepatocellular carcinoma cell lines. (A) Cytotoxicity of ESPS on HepG2.2.15 cells. (B) Cytotoxicity of ESPS on Huh7 cells. HepG2.2.15 or Huh7 cells were incubated with ESPS at indicated concentrations for 48 h. Then, cell viability was measured by the CCK8 method. Values are means ±S.D. (n = 3). * $P < 0.05$ vs. the normal control group.
